# Abstract of the 18th Annual Congress of the SBRA, Salvador, BA, 20-23 August 2014

**DOI:** 10.5935/1518-0557.20140016

**Published:** 2014

## P-01. Comparison of Results from Fresh and Thawed Embryos Taken to Sequential Culture with Transfer Between D5 and D6 of Development

**C.P.M. Teixeira^1^, D.P. Freitas^1^, L.D.T Queiroz^1^, M.S. Borges^1^, I.S. Rocha^1^, G. Coelho^1^**

*^1^Instituto Valenciano de Infertilidade - IVI Salvador*

**OBJECTIVE:** Compare the results of fresh and thawed embryos and that were taken to CS (sequential culture) to transfer between D5 and D6 in a clinic for Human Reproduction in the period of January to May 2014.

**MATERIAL AND METHODS:** We present the results of transfers of 44 patients with a mean age of 36.5 years and varied problems of infertility that transferred blastocysts after sequential culture. We separated two groups of cases: the group of fresh embryos (n = 26) and the group of patients who had cryopreserved embryos in D2 or D3 of development, who were not successful in the first transfer and returned to the center to perform embryo thawed transfer after CS (n = 18).

**RESULTS:** The group of patients with fresh embryos achieved 57.69% (n = 15) of pregnancy, whereas the group of patients with thawed embryos achieved 44.44% (n = 8) of pregnancy. The survival rate of embryos after thaw was 100%.

**CONCLUSIONS:** Our results showed no statistically significant difference between pregnancies obtained in both groups. With efficiency of the techniques of embryo vitrification and thawing is possible to obtain similar to fresh transfer results after sequential culture up to blastocyst of cryopreserved at D2 and D3 embryo, increasing the success rates in patients seeking a second chance of pregnancy after cycles with negative results.

## P-02. Psychology Intervention in Waiting Room of a Public Hospital

**T.E.F. Böhm^1^, G.V. Lourenço^1^, E.P. Passos^1^**

*^1^Hospital de Clínicas de Porto Alegre*

**INTRODUCTION:** This experience report is the result of psychology’s practice traineeship in a public hospital in the metropolitan region of Porto Alegre/RS. For six months (August-December 2013) in the waiting room of Assisted Fertilization sector, women and/or couples who were undergoing treatment for the procedure were followed. The objective was the establishment of a connection, listening, acceptance, restraint, reframing the speech, feelings, anxieties and fears.

**CASES REPORT:** Over the meetings in the waiting room, the place became a setting kind of different since flocked to that spot the most different emotional demands. Expressions of relief were seen, with the presence of psychology professional the waiting room, doing the listening. Fears of judgment were giving courage to expose their anxieties and discontents, their sorrows and despair in the context of loss and mourn for the inability to gestate. The methodology was to encourage patients to put into words the meaning of the experienced situation. Furthermore, to strengthen the defenses, reflect on fantasies, idealizations and coping strategies of independent problem of positive pregnancy outcome.

**COMMENTS:** We observed that the psychological aspects of the patients in the waiting room were being worked out and according to the reports of the medical staff, the approach to psychology has brought benefits. During the conversations of the waiting room, there was the need to strengthen the self-esteem of women and proof the results of assisted fertilization. Previous the procedures was ministered a course for infertile couples. As a preventative measure to strengthen self-esteem, we present a video testimony of successful fertilization and other video on the self-image that every woman has of herself. The presence of the psychology student in the waiting room of assisted fertilization, allowed access to content that will only be accessed and manifested in practice in psychology. Infertility has psychological impacts, as well as stressors that require a holding space.

## P-03. Utilization of the CG-EM Technique for Metabolic Analysis and Embryonic Classification in 3-Day Culture Embryos

**A.K. Bartmann, V.C.P.S. Nascimento, S.H.T. Contini, C.D. Bartolomeu, D.F.R. de Souza, L.L.M. da Silva**

*Clinica Ana Bartmann - Centro de Reprodução Humana UNAERP- Universidade de Ribeirão Preto-SP*

**OBJECTIVE:** To propose a new embryonic classification methodology for day 3 culture (D3) through metabolic parameters, using the GC-MS (Gas chromatography coupled with mass spectrometry) and thereby ascertaining the existing relationship between morphological and metabolic criteria for embryonic classification.

**MATERIAL AND METHODS:** Individual culture media of 3-day embryos were analyzed according to their metabolic profile and compared to the control medium (microdrop in culture with no embryo). Embryos were also photographed and classified according to Veeck’s morphological criteria on day 3. Culture media were processed and the metabolites present were extracted using the silanization and derivatization technique. After extraction, the compounds were analyzed in a gas chromatograph coupled with a mass spectrometer. Pyruvate, lactate, and glucose levels were assessed in all microdrops, including control microdrops. The values of the metabolites found were compared with those of the control drops to assess each embryo’s production/consumption. A metabolic classification table was proposed according to each embryo’s metabolic profile: Class A: high metabolic rate embryo; Class B: alteration in the production or consumption in only one metabolite; Class C: alteration in the production or consumption in two metabolites; Class D: alteration in the production or consumption in the three metabolites studied. The classification of metabolic activity was compared to the morphological classification (photography).

**RESULTS:** Of the embryos classified and analyzed so far, there was an agreement of metabolic and morphological criteria in 32% of the cases.

**CONCLUSIONS:** Aiming to improve pregnancy and livebirth rates, our center uses the sum total of 2 parameters at the time of embryo selection for intrauterine transfer. Additional research is still needed to assess whether or not metabolic criteria are superior to morphological criteria, and whether they should be used together as we do in our clinic.

## P-04. Analysis of the Presence/Absence of the GSTM1 Gene Polymorphism in Blood Samples and Biopsy in Women with Endometriosis

**R.C.P. Costa e Silva^1^, L. Guillo1, K.K.V.O. Moura^2^**

*^1^Universidade Federal de Goiás - UFG*

*^2^Pontifícia Universidade Católica de Goiás - PUC Goiás*

**OBJECTIVE:** The objective of this study was to analyze the presence/absence of the GSTM1 gene polymorphism that could be related to the pathology of endometriosis, in two groups of samples from patients with the disease; and a group of blood and other analysis of the biopsy.

**MATERIALS AND METHODS:** we analyzed 52 women between the ages of 25 to 35 years, met at the centro de referência em videolaparoscopy and infertility. All patients were submitted to videolaparoscopy for the confirmation of the presence of endometriosis. Genomic DNA was isolated from peripheral blood lymphocytes and biopsy samples collected at the same moment of videolaparoscopy. The DNA samples were subjected to PCR amplification, aimed at detection of polymorphism GSTM1. The product obtained by each reaction was subjected to electrophoresis in agarose gel 2%, in an electric field of 10 V/cm stained with ethidium bromide (5µg/L) and the visual record of the gel made with the aid of a video system-documentation.

**RESULTS:** The genotypic frequency GSTM1 found in patients diagnosed with endometriosis in blood sample (n = 47) was 51% (24/47) and GSTM1 null or absent was 49% (23/47); on biopsy sample (n = 52) was found present in 42.3% (22/52) and the null 57.7% (30/52).

**CONCLUSIONS:** the presence of the GSTM1 gene polymorphism in the analysis in the blood of patients with endometriosis was greater than in the biopsy. The absence or invalidity of the GSTM1 gene polymorphism is increased in the biopsy compared to blood.

## P-05. A Comparison of Implantation, Miscarriage and Pregnancy Rates of Day 3 Embryos Transfer Between Blastocyst Embryo Transfer: A Retrospective Study

**J.F. Macedo^1^, L. M. O. Gomes^1^, K.R.B. Melo^1^**

*^1^Clínica Reproferty - São José dos Campos - SP - Brazil*

**OBJECTIVE:** Studies show that culture of embryos to blastocyst stage for subsequent transfer yields high pregnancy rates and minimizes the risk of multiple gestations. The objective this study is to compare the clinical outcomes embryo transfer in day 3 versus blastocyst embryo transfer in selected patients.

**MATERIALS AND METHODS:** This study involved a consecutive series of 120 cycles in women age up to 49 years who are undergoing their first or second trial of in vitro fertilization-embryo transfer (IVF-ET) from January 2007 to December 2012. Were analyzed in the group day 3 and day 5 the following rate: success (embryo number implantation versus total embryos transfer), rate miscarriage and cancellation, and also performed a comparison by age group. Statistical analysis was performed with Biostat 5.0 program, the Anova test was used, following Bonferroni’s test. Results were considered statistically significant if *P* < 0.05.

**RESULTS:** Implantation rate of embryos in relation to the number of embryos transferred were in day 3: 7.23% and day 5: 15.87%. The rate miscarriage in day 3: 92.8% and day 5: 84.2%. The rate cancelation cycle was in day 3: 66.08% and day 5: 34.18%. The present study demonstrated higher success rate in patients aged up to 30 years (blastocyst: 50% rate success and day 3: 11.36%), group 31-35 years age (blastocyst: 14.28% rate success and day 3: 6.77%), group 36- 39 years age (blastocyst: 11.11% rate success and day 3: 3.45%), and in patients aged between 40 and 50 age (blastocyst: 0% and Day 3: 4.34%) showed no significant differences.

**CONCLUSIONS:** In conclusion, our study shows that transfer of blastocyst embryos in women under 30 years of age showed better results than transfer in day 3. For the age group between 31 and 35 years of age and 36-39 years of age there were also significant differences between these two groups but there were no differences in the group 40-49 years of age.

## P-06. Influence in Implantation Rate of Blastocyst Cultured in 5% CO_2_, 5% O_2_ and 90% N_2_ Incubator

**J.F. Macedo^1^, L. M. O. Gomes^1^, K.R.B. Melo^1^**

*^2^Clínica Reproferty- São José dos Campos - SP - Brazil*

**OBJECTIVE:** It was reported in previous studies that either use of a low concentration atmosphere oxygen or addition of antioxidants to the culture media is effective in supporting bovine embryo development in vitro. Low oxygen concentration promoting effect in culture environment of the embryo development and has been reported for the mouse, rabbit and human. In other study were observed whether a change in gas atmosphere promotes development in vitro derived bovine embryos. Thereby the aim is influence evaluate in implantation rate of human embryos cultured in different incubators until blastocyst stage.

**MATERIALS AND METHODS:** This is a retrospective study conducted between January 2009 and December 2012. The study was conducted with 518 patients infertile from which 4 or more oocytes were retrieved. Were analyzed cases cultured in 5% CO_2_ in air incubator until day 3, following cultured in 5% CO_2_, 5% O_2_ and 90% N_2_ until blastocyst stage, control group only cultured in 5% CO_2_ in air incubator. Then were analyzed rate of implantation of embryos in day 5 from different incubators. Statistical analysis was performed with Biostat 5.0 program, the Anova test was used, following Bonferroni’s test. Results were considered statistically significant if *P* <0.05.

**RESULTS:** In the culture embryo using 5% CO_2_, 5% O_2_ and 90% N_2_ obtained efficient development to the blastocyst stage, the results showed which in incubator 90%N_2_ there 31.25 % implantation rate success and in 5% CO_2_ air incubator 19.95% success (control group), were found statistics significant differences with *P* <0.05.

**CONCLUSIONS:** The study showed that implantation rate of embryos is better transferred into blastocyst and cultured in 5% CO_2_, 5% O_2_ and 90% N_2_ incubator. These data corroborate with the literature.

## P-07. Women of 40 Years or More: Success in IVF?

**L. Okada^1^, R. Azambuja^1^, D. Kvitko^1^, A TaglianiRibeiro^1^, A. Petracco^1^, M. Badalotti^1^**

*^1^Fertilitat - Centro de Medicina Reprodutiva - Porto Alegre - Brasil*

**OBJECTIVE:** To assess the rates of pregnancy and abortion in women higher than or equal 40 years old, undergoing IVF.

**MATERIAL AND METHODS:** Retrospective study of 624 couples undergoing IVF between January 2010 to May 2014. The frequency of non-transfer and rates of clinical pregnancy and miscarriage were analyzed by Chi-square test (*P* <0.05).

**RESULTS:** Among the 624 patients, 80 (12.8%) did not undergo embryo transfer for: ruptured follicles at the time of aspiration, lack of aspirated oocytes, absence of mature oocytes, total failure of fertilization or poor embryo development. The patients were divided according to ages: 40 (n = 160), 41 (n = 158), 42 (n = 111), 43 (n = 84), 44 (n = 53), 45 (n = 29) and 46-49 (n = 29) years.

The rates of not transfer according to ages were 6.88%; 8.23%; 9.0%; 26.2%; 11.32%; 31% and 31.0% respectively.

The clinical pregnancy rates were 32.9% (49/149); 30.3% (44/145); 18.8% (19/101); 17.7% (11/62); 8.5% (4/47); 5.0% (1/20); and 0.0% (0/20); respectively. Abortions observed between the groups with clinical pregnancy were 36.7%; 38.6%; 26.3% 81.8%; 75% and 0%; respectively. Patients with 40 years old had 19 births and 12 more pregnancies in progress; at 41, 23 births and 4 in progress; at 42, 13 births and 1 in progress; at 43, 1 and 1 delivery in progress; at 44, one childbirth; and at age 45, 1 delivery.

**CONCLUSIONS:** According to this group, women aged 40 to 41 years old showed similar clinical pregnancy rate; as well as at 42 and 43 years. From 42 years, the rate of clinical pregnancy was statistically significant fall. From 46 years onwards, no pregnancy was observed and abortions rose significantly from 43 years. It is worth mentioning the birth obtained at age 44 and another at 45. Physiologically, after 40 years old, the chance of spontaneous pregnancy drops to 5% per month.

Considering this fact, we conclude that in vitro fertilization in this group had success with pregnancies in women up to 42 years, and successfully taking baby home.

## P-08. Transfer of Frozen Embryos: 1 or 2 Blastocysts?

**V. Reig^1^, L. Proença^1^, R. Azambuja^1^, L. Okada^1^, A. Petracco^1^, M. Badalotti^1^**

*^1^Fertilitat - Centro de Medicina Reprodutiva - Porto Alegre - Brasil*

**OBJECTIVE:** To compare pregnancy rates between patients who underwent transfer of one or two frozen embryos at the blastocyst stage.

**MATERIAL AND METHODS:** During the years 2013 to 2014, 182 cycles of frozen embryos at the blastocyst stage were performed. Of these 51 cycles was only 1 embryo transfer (group 1) and 131 cycles in 2 embryos (Group 2) were transferred. All embryos were cryopreserved by vitrification method Kuwayama et al. (1998). The embryo transfers were performed under ultrasound control, and all patients were maintained with 600 mg of progesterone daily for 12 weeks when the resulting β-hCG was positive. Pregnancy rates of the two groups were compared by chi-square test (*P* <0.05).

**RESULTS:** The mean age of patients in group 1 was 34.8 years, and in group 2 was 34.6 years. The pregnancy rate in group 1 was 35.3%, and 29.4% (15/51) of clinical pregnancy. In group 2, the pregnancy rate was 52.7%, and 43.5% (57/131) clinical pregnancy. No statistical difference between groups (*P* = 0.0928) was observed. Twin pregnancies occurred in 14 transfers in the group where 2 blastocysts (24.56%) were transferred.

**CONCLUSIONS:** We found that the pregnancy rate is not significantly influenced when transferring one or two embryos occurs; however, only in group 2 twin pregnancy was observed. Thus, it is suggested that the transfer of a single blastocyst decrease the incidence of twin pregnancies without affecting the clinical pregnancy rate. Similar results were found by our group when we compared the transfer of one or two fresh blastocysts.

## P-09. The Impact of Food Intake and Social Habits on Embryo Quality and the Likelihood of Blastocyst Formation

**G. Halpern^1^, D.P.A.F Braga^1,2^, A.S. Setti^1,2^, R.C.S. Figueira^1,2^, A. Iaconelli Jr.^1,2^, E. Borges Jr.^1,2^**

*^1^Fertility - Centro de Fertilização Assistida*

*^2^Instituto Sapientiae - Centro de Estudos e Pesquisa em Reprodução Assistida*

**OBJECTIVE:** To evaluate whether patients’ lifestyle factors and eating habits can influence embryo quality, the likelihood of blastocyst formation and intracytoplasmic sperm injection (ICSI) outcomes.

**MATERIAL AND METHODS:** The study included 1705 embryos recovered from 269 patients undergoing ICSI cycles between January 2012 and July 2013. All patients completed a questionnaire with multiple-choice questions prior to the beginning of the treatment. The women were asked about the frequency of their consumption of many food items and about their social habits. The effects of dietary and social habits on embryo quality on day three and the likelihood of blastocyst formation were evaluated. Moreover, the influence of dietary and social habits on pregnancy and miscarriage rates was also investigated.

**RESULTS:** The consumption of cereals (OR: 1.34, *P*=0,019), vegetables (OR: 1.25, *P*=0.025) and fruits (OR: 1.38, *P*=0.018) positively influenced the embryo quality at the cleavage stage. The quality of the embryo at the cleavage stage was also negatively correlated with the consumption of alcoholic drinks (OR: 0.75, *P*=0.032) and smoking habits (OR: 0.95, *P*=0.044). The consumption of fruits influenced the likelihood of blastocyst formation (OR: 1.31, *P*=0.08), which was also positively affected by the consumption of fish (OR: 1.32, *P*=0.018). Being on a weight-loss diet (OR: 0.78, *P*=0.046), the consumption of red meat (OR: 0.81, *P*=0.049), and alcoholic drinks (OR: 0.75, *P*=0.032), as well as smoking habits (OR: 0.76, *P*=0.035) had negative influences on the likelihood of blastocyst formation. Being on a weight-loss diet (OR: 0.79, *P*=0.011), the consumption of red meat (OR: 0.68, *P*=0.042) and the body mass index (OR:0.43, *P*=0.046) had a negative impact on the likelihood of pregnancy. The occurrence of miscarriage was not influenced by any food consumption or social habit.

**CONCLUSIONS:** Our evidence suggests a possible relationship between environmental factors and ovary biology. Therefore, couples seeking assisted reproductive technology must be advised about the adverse effects of female lifestyles on treatment success.

## P-10. Human Sperm Lipid Fingerprinting by Mass Spectrometry - A Pilot Study

**A.S. Setti^1,2^, T. Serzedello^1^, E. Lo Turco^3^, E. Cabral^4^, M. Eberlin^4^, E. Borges Jr.^1,2^**

*^1^Fertility - Centro de Fertilização Assistida*

*^2^Instituto Sapientiae - Centro de Estudos e Pesquisa em Reprodução Assistida*

*^3^Universidade Federal de São Paulo - UNIFESP*

*^4^ThoMSon Mass Spectrometry Laboratory*

**OBJECTIVE:** To identify a prognostic tool of sperm quality by correlating the lipid profile with the male age, sperm DNA fragmentation and MSOME by using the analytical power of mass spectrometry (MS).

**MATERIALS AND METHODS:** Semen samples from 27 patients undergoing ICSI were analysed by MSOME, DNA fragmentation test and lipid profile. Samples were divided in quartiles according to patient’s age, DNA fragmentation index and incidence of sperm with large nuclear vacuoles (LNV). For the lipid profile, each sample was analysed in triplicates. Mass spectra fingerprinting were acquired using a 7.2T LTQ FT Ultra-MS, equipped with a chip-based direct infusion nanoelectrospray ionization source. Data were analysed using the Partial Least Square Discrimination Analysis (PLS-DA) combined with variable influence on projection (VIP) scores. The lipid profiles were compared between the different groups of male age, MSOME and sperm DNA fragmentation results.

**RESULTS:** Following the VIP analysis, the most important lipid species were detected. Regarding male age, the model detected a significant increase of 4 lipids in the 1st quartile group (range: 28-32 years), 6 lipids in the 2nd quartile group (range: 33-36 years), 4 lipids in the 3rd quartile group (range: 37-42 years) and 1 lipid in the 4th quartile group (range: 43-54 years), with an accuracy of 60.0%. As for DNA fragmentation index, the model detected an increase of 5 lipids in the 1st quartile group (range: 7.7-12.5%) and 10 lipids in the 4th quartile group (range: 18.5-31.5%), with an accuracy of 84.6%. Regarding the incidence of LNV sperm, the model detected a significant increase of 5 lipids in the 1st quartile group (range: 2-6%) and 10 in the 4th quartile group (range: 22-42%), with an accuracy of 85.0%.

**CONCLUSIONS:** Specific lipids that are differentially represented depending on the male age, sperm DNA fragmentation index and incidence of LNV sperm were detected. The MS fingerprinting may be a valuable, non-invasive tool for the prediction of semen sample quality.

## P-11. The Efficiency of a Donor-Recipient Program using Infertile Couples’ Egg Cryo-Banking: A Brazilian Reality

**R.C.S. Figueira^1,2^, A.S. Setti^1,2^, D.P.A.F Braga^1,2^, A. Iaconelli Jr.^1,2^, E. Borges Jr.^1,2^**

*^1^Fertility - Centro de Fertilização Assistida*

*^2^Instituto Sapientiae - Centro de Estudos e Pesquisa em Reprodução Assistida*

**OBJECTIVE:** To explore if the Brazilian egg-donation treatments’ outcomes obtained with cryopreserved oocytes donated from infertile patients are equivalent to those obtained worldwide with cryopreserved oocytes donated from fertile egg-donors.

**MATERIALS AND METHODS:** From January 2009 to July 2013, oocytes were obtained from patients undergoing ICSI who decided to donate their surplus oocytes. Vitrification and warming procedure were performed using the Cryotop method. We described the survival, fertilization, blastocyst, implantation and pregnancy rates obtained in our infertile donor-recipient program. We compared the results obtained in our infertile donor-recipient program with the results from published studies. To that end, we used the critical review of the literature performed by Edgar and Gook et al 2012. We also performed a search in Pubmed to identify if any more articles were published.

**RESULTS:** A total of 259 egg donation cycles, with 1622 donated oocytes, were performed in our donor-recipient program, with a post-warm survival rate of 77.1% (1251/1622). We obtained a fertilization rate of 72.9% (874/1199), a blastocyst formation rate of 53.8% (465/874), an implantation rate of 31.1% (110/354) and a pregnancy rate per warmed oocyte of 5.4% (87/1622). We analyzed seven studies dealing with cryopreserved egg-donation. The studies, performed between 2008 and 2013, included varying numbers of egg-donors (range: 20-600), warmed oocytes (range: 123-3826) and survival rates (range: 85.6-92.5%). Fertilization rates ranged from 74.2% to 87.0%, blastocyst formation rates from 41.3% to 68.0%, implantation rates from 24.7% to 55.3% and the estimated clinical pregnancy rate per oocyte ranged from 3.9% to 9.8%.

**CONCLUSIONS:** Our Brazilian experience of freezing surplus eggs from IVF cycles of infertile couples yields satisfactory results that are not disparate from other reported presumed fertile egg donors, related to survival, fertilization, embryo development and pregnancy rates.

## P-12. Sperm Morphological Abnormality under high Magnification Predicts Embryo Development, from Fertilization to the Blastocyst Stage, in Couples Undergoing ICSI

**E. Borges Jr^1,2^, A.S. Setti^1,2^, L. Vingris^1^, D.P.A.F Braga^1,2^, R.C.S. Figueira^1,2^, A. Iaconelli Jr.^1,2^**

*^1^Fertility - Centro de Fertilização Assistida*

*^2^Instituto Sapientiae - Centro de Estudos e Pesquisa em Reprodução Assistida*

**OBJECTIVE:** To study the onset of the detectable paternal effect on embryo development by associating the sperm quality under motile sperm organelle morphology examination (MSOME) and embryo morphology.

**MATERIAL AND METHODS:** A total of 376 embryos obtained from 60 couples undergoing ICSI in a private assisted fertilization center had their morphology evaluated on days 1, 2, 3 and 5 and associated with the percentage of sperm with large nuclear vacuoles (LNV) identified through MSOME. Binary regression analyses were performed to study the influence of LNV sperm on embryo quality, from pronuclear to blastocyst stage. Receiver operating characteristic (ROC) curve analysis was performed to assess the predictive value of LNV spermatozoa on the development of a blastocyst. The Youden index was used to enable the selection of an optimal threshold value for the incidence LNV sperm.

**RESULTS:** The percentage of LNV sperm was determinant to the increased odds of a zygote presenting pronuclear abnormalities. It was also determinant to the decreased odds of (i) normal cleavage on days 2 (OR: 0.76) and 3 of embryo development (OR: 0.75), (ii) an embryo showing high-quality on day 3 of development (OR: 0.96), (iii) an embryo develop to the blastocyst stage (OR: 0.90), and (iv) an embryo showing a normal trophectoderm (OR: 0.84) and inner cell mass (OR: 0.85). The areas under the curves (AUC) distinguished between embryos that did develop to the blastocyst stage or not (AUC: 0.673, *P*<0.001) and blastocysts of high- or low-quality (AUC: 0.766, *P*<0.001). The best cut-off for the percentage of LNV sperm that maximizes is ≤ 24.5% for blastocyst formation and ≤ 19.5% for high-quality blastocyst.

**CONCLUSIONS:** The reduced zygote and embryo quality, and blastocyst formation and quality associated with poor semen morphology under high-magnification suggest a very early onset of paternal effects on embryo development. The evaluation of sperm vacuoles by MSOME may significantly upgrade the prognostic information provided by conventional semen analysis.

## P-13. The Influence of the Concentration of Spermatozoa on the Outcome of Intrauterine Insemination at a Service of Human Reproduction in 2013 - Cross Study

**R.S.A. Sasaki^1^, M.S. Approbato^1^, M.C.S. Maia^1^**

*^1^Centro de Reprodução Humana do Hospital das Clínicas da Universidade Federal de Goiás*

**OBJECTIVE:** Assess whether couples whose partner with sperm concentration less than 60 million/ml have a higher chance of pregnancy than those with concentrations between 15 and 59 million/ml in intrauterine insemination.

**MATERIAL AND METHODS:** A retrospective crosssectional study. Files of 63 couples undergoing intrauterine insemination were evaluated in 2013, and the couple divided into two groups: those whose partner had sperm concentration of between 15 and 59 million/ml (group 1) and those whose partner had sperm concentration above 60 million/ml (group 2). The variables analyzed were: age of man and woman, sperm concentration, percentage of sperm sum of grade A and B, total processed after capacitation of spermatozoa and the presence or absence of pregnancy after intrauterine insemination sperm. Statistical analyzes were performed descriptive and the presence of laboratory pregnancy between groups was submitted to the X^2^ test, using the SPSS 17 program.

**RESULTS:** Group 1 consisted of 30 couples, with a mean age of 34.15 years for men and 33.00 years for women. The concentration of sperm had an average of 34.5 million/ ml. The sum of the sperm grade A and B was 49.51% on average. The average of total processed spermatozoa (TEP) was 16.00 million/ml. Group 2 consisted of 33 couples, with a mean age of 36.70 years of man and 33.90 years for woman. The average concentration of spermatozoa was 98.57 million/ml. The sum of the sperm A and B was 58.33% on average. The average T.E.P. was 28.50 million/ml. The success rate of pregnancy represented by the laboratory was 19.5%, and in group 1 and 23.33% in group 2 of 15.15%. The X^2^ test was 0.68 and *P*=0.41, not significant at *P*<0.05.

**CONCLUSIONS:** The success rate represented by the pregnancy was not statistically different between couples whose partner had sperm concentration between 15 and 56 million/ml and those with greater than or concentration greater than or equal to 60 million/ml.

## P-14. Estradiol On the Second Day is not Related to Pregnancy Rate

**T.V. Oliveira^1^, D. Souza^1^, A.C. Azevedo^1^, F.C. Miyasato^1^, M.C.R.M. Albuquerque^1^, L.E.T. Albuquerque^1^**

*^1^Centro de Reprodução Humana FERTIVITRO*

**OBJECTIVES:** The real importance of estradiol levels on the second day of the menstrual cycle in IVF results remains controversial. Up to now, there are few studies in the literature and there is no consensus among the authors if high level of estradiol on the beginning of the cycle reduces the chances of pregnancy. The importance to know if this relationship exist is, in fact, to analyze the possibility to start or cancel an IVF cycle when estradiol is above 75 ng / mL. This study aims to assess if the levels of serum estradiol on the second day of the menstrual cycle is correlated with pregnancy rates in IVF cycles (IVF).

**MATERIAL AND METHODS:** We retrospectively analyzed 102 patients undergoing IVF technique at the Human Reproduction Center FERTIVITRO, from June 2011 to June 2012, where blood samples were collected on the second day of the menstrual cycle to evaluate serum levels of estradiol. The groups were stratified by age. Group I: ≤ 37 years. Group II: ≥ 38 years. The blood samples of all patients were collected and the estradiol levels were analyzed and compared between pregnant and non-pregnant patients of each group.

**RESULTS:** In Group I the estradiol concentration average was 51.67 ng / ml (± 16.43), and in the pregnants patients was 51.56 ng / ml (± 16.5). In Group II, the estradiol concentration average was 49.21 ng / ml (± 22.14) and in the pregnant patients was 48.29 ng / ml (± 17.68). The abortion rate was 25.6% when the estradiol concentration average was 48.35 ng / ml (± 6.05) and the average age was 36 years (± 5.4).

**CONCLUSIONS:** Our results showed no statistical difference on the serum levels of estradiol in pregnant and non-pregnant patients. Due to this, we can believe that the plasma estradiol level on the second day of the menstrual cycle does not predict success or failure in attempts of IVF and also we not observed relationship with the abortion rates.

## P-15. Comparison Conventional PVP-ICSI Vs. HA-PICSI: Is There Significant Improvement in Pregnancy Rate?

**T.V. Oliveira^1^, A.C. Azevedo^1^, F.C. Miyasato^1^, L.S. Fujihara^1^, M.C.R.M. Albuquerque^1^, L.E.T. Albuquerque^1^**

*^1^Centro de Reprodução Humana FERTIVITRO*

**OBJECTIVE:** In nature, human oocytes are surrounded by Hyaluronic Acid (HA). In fact, only mature spermatozoa which have extruded their specific receptors to bind to and digest HA can reach the oocyte and fertilize it. Up to now, different methodologies to select sperm have been described in the hope of selecting a healthy sperm for use with ICSI. Usually this selection is based on sperm morphology and motility but is not discriminatory with respect to the identification of spermatozoa with normal haploid chromosome, intact chromatin or DNA which reduces chromosomal imbalance risks. The aim of this study was to compare the results in many parameters of the same patients that realized treatment by conventional PVP-ICSI and HA-PICSI.

**MATERIALS AND METHODS:** We retrospectively study 10 couples undergoing IVF treatment from 2010 to 2012 that realized a conventional PVP-ICSI first and have no success, after that realizes HA-PICSI. The parameters analyzed were: average age, sperm concentration, sperm motility, number of mature oocytes, fertilization rate, embryo quality and pregnant rate. The statistical analysis was performed by SPSS program (Statistical Package for Social Science) in order to verify possible differences among the parameters above between PVP-ICSI and HA-PICSI.

**RESULTS:** This study showed statistical difference in the man and women average age when we compared the same couple who realize a cycle using conventional PVP-ICSI with no success and after that trying again using HA-PICSI (37.6±4.9 x 38.6±5.0 and 35.2±3.9 x 36.2±3.8 respectively; *P*=0.014). However, we did not have statistical differences between the others parameters evaluated (sperm concentration, sperm motility, number of MII, fertilization rate and embryo quality). But, the pregnancy rate was 20% with conventional PVP-ICSI x 80% with HA-PICSI and the abortion rate was 100% and 12.5% respectively (*P*<0.050).

**CONCLUSIONS:** In this study we can conclude that the use of HA-PICSI technique can improve the pregnancy rate and decrease abortion rate even when the couple´s age was higher leading to believe that HA-PICSI technique can help us to choose a mature spermatozoa with intact DNA. Despite this, we need more prospective randomized studies to confirm the efficacy of HA-PICSI method.

## P-16. Association of Translocation X-Autosome T (X; 11) (Q22, Q21) with Premature Ovarian Failure

**M. Santos^1^, E.B. Cordts^1^, F.A. Mafra^1^, B. Bianco^1^, C.P. Barbosa^1^, D.M. Christofolini^1^**

*^1^Center Of Human Reproduction and Genetics - Department of Human Reproduction - Faculdade de Medicina do ABC, Santo André / SP, Brazil*

**INTRODUCTION:** Premature Ovarian Failure (POF) is an ovarian dysfunction, characterized by cessation of menstruation resulting in anticipation of menopause. POF affects about 1 in every 100 women in the population between 30-39 years old. This ovarian dysfunction may be secondary to several factors, among them balanced X-autosome translocations. There is a specific region on the long arm of the X chromosome that is associated with the formation of the female gonad and maintenance of ovarian function, described as “critical region of Xq” comprising from Xq13 to Xq26. Then, it is believed that the genes present on the Xq chromosome are essential for normal ovarian function.

**CASE REPORT:** The case introduce a woman with 35 years old, married, Brazilian, menarche at 12 years old, with a history of irregular cycles of 05 days with an interval of 06 months that never performed a gynecological surgery. The couple presents 04 years of marriage and 01 years of primary infertility. Two dosage of follicle stimulating hormone (FSH) were performed with a month apart. Besides, cytogenetic analysis from lymphocyte culture and subsequent G banding were performed. The two dosages of FSH and results are 75.5mUI/mL and 98.8mUI/ mL. Karyotype result showed a X-autosome translocation 46,XX,t(X,11)(q22;q21). X chromosome breakage happened in the critical region for POF.

**COMMENTS:** Translocations alter the spatial organization of “critical region of Xq” and may result in dysregulation of the expression of genes located in this region, generating a defect in the formation of the ovaries. Considering assisted reproduction treatment, patients with suspected POF should be evaluated by cytogenetic analysis at the beginning of treatment to prevent miscarriages and implantation failure and should be instructed to perform preimplantation genetic diagnosis (PGD) to attempt selection of normal embryos. Besides the damage reported on infertility, the balanced chromosomal translocations cause a repositioning of chromosomal fragments, leading to changes in global gene expression by affecting the expression of genes that are close, distant or even on other chromosomes and can be associated to neoplasia predisposition.

## P-17. Comparative Study of the Results of Fertilization after ICSI (Intracytoplasmic Sperm Injection) of Fresh and Thawed Oocytes

**D.P. Freitas^1^, C.P.M Teixeira^1^, L.D.T Queiroz^1^, M.S. Borges^1^, I.S. Rocha^1^, G. Coelho^1^**

*^1^Instituto Valenciano de Infertilidade - IVI Salvador*

**OBJECTIVE:** Compare the rate of fertilization of fresh and thawed oocytes undergoing ICSI at a clinic of Human Reproduction in the period of January to May, 2014.

**MATERIAL AND METHODS:** In this study we analyzed the result of fertilization of 303 oocytes in metaphase II (MII) of patients aged <35 years. The patients were divided into two groups: patients with fresh MII oocytes undergoing ICSI (n = 226) and patients with thawed MII oocytes undergoing ICSI (n = 77).

**RESULTS:** The fertilization rate of patients with fresh oocytes MII was 65.48% (n = 148), whereas for the group of patients with thawed oocytes the fertilization rate was 66.23% (n = 51). The overall fertilization rate was 65.67%. No statistically significant difference was observed in fertilization rates between groups of patients with fresh and thawed oocytes. The survival rate of thawed oocytes was 97.46% (total = 79 oocytes).

**CONCLUSIONS:** This study shows that with a good cryopreservation program can be obtained fertilization rates of thawed oocytes similar to those rates of fresh oocytes. With efficiency in the techniques of vitrification and thawing oocytes is possible to maximize results using cryopreserved oocytes for several reasons.

## P-18. Molecular Analysis of Products of Conception by SNP Array

**F. Mafra, L. Kulikowski, B. Bianco, C.P Barbosa, D.M. Christofolini**

*Instituto Ideia Fertil - Laboratório de Genética Reprodutiva Faculdade de Medicina do ABC, Santo André, SP, Brasil*

**OBJECTIVES:** Molecular characterization of genomic alterations present in samples derived from human embryonic curettage through SNP array technique.

**MATERIAL AND METHODS:** Were analyzed 18 samples of product of conception obtained through curettage in the period of June/2011 to February/2014. Samples of maternal tissue, like peripheral blood or swabs of oral cells were also collected for comparison with the embryonic material and to remove possible maternal contamination. Analyses were performed using the SNParray-300K BeadChip (Illumina) in combination with GSN’s Parental Support Technology Bioinformatics considering parental genotype data to interpret the results in the sample tested.

**RESULTS:** Seven samples had normal results (38%), being 2 cases 46,XY and 5 cases 46,XX. However, in two cases of the karyotype 46,XX were identified only maternal cells, without the presence of fetal material. Triploidy was observed in 2 cases (11%), aneuploidy of chromosome 9 (5%), 14 (5%), 15 (5%), 16 (5%), 22 (11%) and one case with two concomitant alterations 45,X,+9. One sample showed mosaicism involving the sex chromosomes (46,XX/46,XY). And finally, one sample showed deletion of the terminal region of chromosome 15 [46,XX,del(15) (q26.2-qter)]. Of the altered cases, 70% of aneuploidy was of maternal origin, 20% of paternal origin and in 10% caused by both parents. The mean age of the pregnant was 36.84 (± 5.14) years. The average of weeks of pregnancy loss was 10.56 (± 5.98).

**CONCLUSIONS:** The SNP array technique is efficient to identify pathogenic genomic alterations, lower than those detected by karyotype, besides identifying the parental origin of the alteration, allowing a rapid diagnosis and making possible a safe conduct of genetic counseling for families.

## P-19. Identification of Polymorphisms on WNT4 Gene in Infertile Patients with Endometriosis

**F. Mafra, B. Bianco, C.P. Barbosa, D.M. Christofolini**

*Instituto Ideia Fertil - Laboratório de Genética Reprodutiva /Faculdade de Medicina do ABC, Santo André, SP, Brasil*

**OBJECTIVE:** Evaluation of polymorphisms (rs16826658, rs7521902, rs3820282, rs2235529) on WNT4 gene in infertile patients with endometriosis and in the control group.

**MATERIAL AND METHODS:** Case-control study comprising 400 infertile women with endometriosis (n=200 endometriosis I/II, n=200 endometriosis III/IV) and 400 fertile woman of the control group (group submitted to tubal ligation, confirming the absence of endometriosis by inspection of pelvic cavity during the laparoscopy). The polymorphisms will be evaluated by Real Time PCR through Taqman methodology.

**RESULTS:** Until this moment, was performed the analysis of the polymorphism rs2235529 revealing the genotypes GG (wild type), GA (heterozygote) and AA (polymorphic), respectively with the frequency of, 72.5%, 24.25% and 3.25% for the endometriosis group. In relation to the control group, the genotypes were GG 77%, GA 21.25% and AA 1.75%. The alleles G and A were present in 84.62% and 15.38% of infertile woman with endometriosis, and in 87.62% and 12.38% of the control group. The chi-squared test revealed no differences in allele and genotype frequencies between patients x controls (*P*=0.3473), not even considering the stage of the disease, minimal/mild endometriosis x control (*P*= 0.1728), moderate/severe x control (*P*= 0.6976).

**CONCLUSIONS:** No difference was found between endometriosis-related infertility and controls in the analysis of the first polymorphism.

**Support:** This work was supported by grants from FAPESP (Fundação de Amparo a Pesquisa do Estado de São Paulo) # 2013/14470-9

## P-20. Case Report: Couple Carrying the Gene of Niemann-Pick Type C Syndrome, Submitted to Preimplantation Genetic Screening (PGS), gets Pregnant after Transfer of Unaffected Embryos

**G. Coelho^1^, I.S. Rocha^1^, D.P. Freitas^1^, C.P.M. Teixeira^1^, L. D.T. Queiroz^1^, M. Borges^1^**

*^1^Instituto Valenciano de Infertilidade - IVI Salvador*

**INTRODUCTION:** Niemann-Pick Syndrome is a neurovisceral rare disease, autosomal recessive, caused by mutations in genes NPC1 and NPC2, can present in children or adults. Neonates can present ascites, liver disease and pulmonary disease. The classic presentation occurs in mid-to-late childhood with hypotonia, developmental delay, ataxia, vertical supranuclear gaze palsy (VSGP) and dementia. Dystonia and seizures are pneumonia. Adults can present dementia or psychiatric symptoms.

**CASE REPORT:** RBP (39 years) and RDP (40 years) carries gene NPC1, G2P2A0, have two daughters with the syndrome. Undergoing treatment of in-vitro fertilization with genetic testing to prevent transmission of the disease. Due to the low ovarian reserve three cycles to accumulation of MII oocytes (metaphase II) were performed, were vitrified: 2, 3 and 5 MII. In three subsequent cycles embryos were vitrified at D2 (day 2 of development) 4, 2 and 6 embryos. All oocytes (n = 10) and embryos (n = 12) were thawed, resulting in 16 embryos left to sequential culture. Biopsy of 6 blastocyst was performed. The blastomeres were sent to CGH-array (Comparative Genomic Hybridization Microarray) and PGD / PCR (Single Gene Diagnosis) for screening of the NPC1 gene. The embryos were vitrified to await results. PCR displayed 5 unaffected embryos with at least 1 standard parental allele. CGH displayed only 2 euploids embryos. Were thawed 2 normal embryos in both analyzes and transferred, resulting positive BHCG with 1 gestational sac. Current pregnancy.

**COMMENTS:** The PGS associated techniques of assisted reproduction and genetic enabling the analysis and treatment of infertility. The CGH identifies gain and loss of chromosomes, but is unable to identify gene mutations and does not prevent hereditary diseases from being transmitted. PGD/PCR reduces the risk of having offspring with this mutations and genetics diseases. The probability of these techniques efficiency is not 100%.In this couple with the results of CGH and PGD/PCR their risk of having offspring with Niemann-Pick Type C were reduced from the expected risk of 25%, to a value significantly less, but that this risk is not reduced to zero.

## P-21. Oocytes with Low Resistance Membrane are

**More Likely to Cellular Death after *In Vitro* Fertilization by ICSI**

**J.A. Lucca^1^, V.B. Rosa^1^, A.P. Peixoto^1^, A. Schuffner^1^**

*^1^Conceber Centro de Medicina Reprodutiva*

**OBJECTIVE:** This work aims to connect the presence of oocyte dismorphism at ICSI with further embryo development.

**MATERIALS AND METHODS:** Were evaluated 662 fresh oocytes, M2, from 136 ICSI cycles between June 2013 and April 2014. The oocyte morphology were classified in: normal (N), granular cytoplasm (GC), large polar body (LPB), large perivitelline space (LPS), low resistance membrane (LRM), oval, aggregates of smooth endoplasmatic reticulum (SER) and vacuolated (VAC). The following embryos were classified according to a top standard criteria defined for each development day. The statistic analysis was performed using the chi-square test with a *P*<0.05 significance.

**RESULTS:** The rate of cellular death after ICSI was higher in morphological abnormal oocytes compared with morphological normal (20.3% and 1.4% respectively; *P*<0.0001), meanwhile the normal fertilization rate (2PN) was similar between the groups (58.1% and 70.7% respectively; *P*=0.86). The rate of top standard embryos was similar between abnormal and normal oocytes in days 2 (58.7% and 64.2% respectively; *P*=0.34) and 3 (45.6% and 50.8% respectively; *P*=0.39). The blastocyst formation rate was similar (28.6% and 34.3% respectively; *P*=0.47) between the groups, however was a tendency to formation of better blastocysts from morphological normal oocytes (13.9% and 20.4% respectively; *P*=0.07). When individually evaluated, the oocytes with LRM had higher cell death rate after ICSI than the normal (51.5% and 1.4% respectively; *P*<0.0001), and than the others dismorfisms.

**CONCLUSIONS:** Despite of several works published, there still no consensus about oocyte dismorphisms consequences at in vitro reproduction cycles. Our study observed a notorious predominance of cell death in oocytes with LRM (51.5%). This morphological mark has relationship with cytoskeleton structure problems, that keeps the cell organization. However, this dismorphism can only be detected at the time of ICSI, which doesn’t allow the exclusion of these oocytes performing a preliminary non-invasive exam. In these cases suggests, if possible, the injection of more oocytes. Further works should be able to correlate this dismorphism with other characteristic in order to avoid injection of oocytes with LRM.

## P-22. Efficiency Evaluation of Assisted Reproducton

**Techniques (Art’s) in Patients with Endometriosis**

**J. Polisseni^1^, P.C. Ribeiro^1^, F. Polisseni^1^, J.V. Rosa^1^, N.F. Oliveira^1^, J.P. Caetano^1^**

*^1^Clínica de Medicina Reprodutiva Pró-Criar/Monte Sinai - Juiz de Fora - MG*

**OBJECTIVE:** To evaluate the efficiency of ART’s (embryo quality and pregnancy rate) in patients who only have endometriosis as a cause of infertility.

**MATERIALS AND METHODS:** A retrospective study was performed under descriptive aspect, through selection of the clinical records in Forest Mining Zone of 2001-2011. Was included in the study only patients with endometriosis as a cause of infertility, age range 26-35 years and who had been treated by Intracytoplasmic Sperm Injection (ICSI) and Intrauterine Insemination (IUI). Embryos were evaluated and classified according to the protocol of the Latin American Network of Assisted Reproduction. The pregnancy rate was assessed by the proportion of pregnant women among the total number of patients who were submitted to TRA’s, each study group. Chi-Square test, with *P*<0.05 was performed.

**RESULTS:** We evaluated 13 IUI cycles and 24 cycles of ICSI. The mean age of patients was 32.3 years. The fertilization rate in ICSI was 84.2% (273/324). After the assessment of embryo quality, 48 hours after fertilization, it was observed that most of the embryos was in the four-cell stage 51.7% (119/234), whereas only 10.7% (25/234), 19.2% (47/234) and 18.4% (43/234) had, respectively two, three and five or more cells (*P* <0.05). 72 hours after fertilization 60.7% (113/186) is found in 3-7 cells, 26.9% (50/186) with eight cells, while 12.4% (23/186) with nine or more cells. No significant difference was observed when evaluated embryo quality embryos 48 and 72 hours (*P*=0.326). The blastocyst rate was 35.3% (24/68). There was no significant difference (*P*>0.05) when the days of embryo transfer and pregnancy rates compared. The rate of ICSI clinical pregnancy was 41.7% (10/24), while the technique of IUI was 7.7% (1/13) *(P*=0.031).

**CONCLUSIONS:** The endometriosis did not affect embryo quality and clinical pregnancy rate using the ICSI technique. However, patients with endometriosis who underwent IUI had lower than expected by the method in this age group of patients (15-20%) results, demonstrating that ICSI seems to be more effective for patients with endometriosis independent of the degree of the disease.

## P-23. Analytical Study of the Results of βHCG (Human

**Chorionic Gonadotropin) Related to the Morphology Of Prepared Semen for Human Artificial Insemination (IAH)**

**L.D.T Queiroz^1^, D. P. Freitas^1^, C.P.M. Teixeira^1^, J.F. Santos^1^, M.S. Borges^1^, G. Coelho^1^**

*^1^Instituto Valenciano de Infertilidade - IVI Salvador*

**OBJECTIVE:** Analyze the results of βHCG related to the morphology of semen that had from 4% normal forms (> 4%), according to WHO (World Health Organization), in the period of January to May 2014.

**MATERIALS AND METHODS:** We compared the results of 39 human artificial inseminations (AHI’s) with patients presenting up to 37 years old. The patients were separated into groups: cases with normal morphology and positive βHCG (n = 4), normal morphology and negative βHCG (n = 8), donor sperm bank with positive βHCG (n = 1), donor sperm bank with negative βHCG (n = 7), group of patients with abnormal morphology and positive βHCG (n = 0) and patients with abnormal morphology and negative βHCG (n = 19).

**RESULTS:** The group of patients with normal morphology (> 4%) achieved 33.33% of gestation, the group of donor sperm bank resulted in 12.5% of gestation, while the group of abnormal morphology (below 4%) achieved 0 % of gestation. The overall pregnancy rate of the 39 cases of AHI’s was 12.8%. Statistically significant difference was observed in the rates of pregnancies related to morphology.

**CONCLUSIONS:** The result of this study demonstrates the importance of the morphology of semen when choosing the appropriate treatment for each couple. Thus indicating the initial examination of sperm analysis for each patient before choosing and start treatment for infertility.

## P-24. Endometrial Scratching in Patients with Recurrent Implantation Failure: A Case Series Study

**L.M. Coutinho^1^, F. Polisseni^1^, J.V. Rosa^1^, J. Polisseni^1^, J.P.J. Caetano^1^**

*^1^Clínica de Medicina Reprodutiva Pró-Criar Juiz de Fora*

**INTRODUCTION:** The endometrial scratching is a mechanical endometrial injury, made in the cycle preceding the embryo transfer in In Vitro Fertilization (IVF). This injury can be induced either by endometrial biopsy using pipelle, or by the hysteroscopy tube. It has been proposed to improve IVF outcomes in woman with unexplained recurrent implantation failure. In theory, this endometrial manipulation could lead to an intense inflammatory response, with a massive secretion of growth factors and cytokines during the healing process, able to optimize the process of embryo implantation.

**CASES REPORT:** Between July 2012 and April 2014, endometrial injury was performed in 17 patients with a history of, at least, two previous implantation failures. The mean age was 34.7 years. Two of these women underwent endometrial scratching and subsequent embryo transfer twice, so 19 procedures were performed. The endometrial biopsies were done with pipelle. Of 19 endometrial scratching, only 3 (15.8%) were done in the preceding cycle of ovarian stimulation and the transfer was made with fresh embryos. In the other cases (84.2%), injury was performed in the menstrual cycle prior to frozen-thawed embryo transfer. The average number of embryos transferred per patient was 2.3. The percentage of women underwent blastocyst transfer was 89.4% (17/19). The remaining 10,6% (2/19) underwent embryo transfer on day 3.

The treatment resulted in pregnancy in 29.4% of patients (5/17). The pregnancy rate per transfer was 26.3% (5/19).

**COMMENTS:** The process of embryo implantation remains a challenge for IVF outcomes. As a retrospective study, with a limited population number and the most cases performed after cryopreservation, this series cases does not have intention to recommend or admonish de use of endometrial scratching in routine clinical practice. Large randomized trials are required in order to confirm the benefits of this practice in woman with recurrent implantation failure.

## P-25. Elective Embryo Transfer after Vitrification: A Case Series Study

**F. Polisseni^1^, J.V. Rosa^1^, J. Polisseni^1^, L.M. Coutinho^1^, J.P.J. Caetano^1^**

*^1^Clínica de Medicina Reprodutiva Pró-Criar Juiz de Fora*

**INTRODUCTION:** The cryopreservation of all embry os followed by a frozen-thawed transfer is often the best option in some patients undergoing in vitro fertilization (IVF) cycle, as women at risk of ovarian hyperstimulation syndrome (OHSS) or patients with inadequate endometrial thickness. Because of important advances in the techniques of embryo cryopreservation, it is possible, nowadays, to postpone embryo transfer, without compromising FIV outcomes.

**CASES REPORT:** This case series study reports our initial experience with cryopreservation of all embryos, without fresh transfer, in IVF cycles. Between February 2012 and March 2014, 8 patients undergoing intracytoplasmic sperm injection (ICSI) did not experienced fresh embryo transfer for one the following reasons: 1) 37,5% risk of OHSS (3/8); 2) 25% inadequate endometrial thickness during ovarian stimulation (2/8); 3) 37,5% oocyte recipients without endometrial preparation for a fresh embryo transfer (3/8). Each patient underwent up to 3 transfers, totaling 12 procedures. The mean age of patients undergoing oocyte retrieval was 35.4 years. The average number of embryos transferred per patient was 2.75, in a total of 33 embryos. The percentage of women underwent blastocyst transfer was 58.3% (7/12). The remaining 41.7% (5/12) underwent embryo transfer on day 3. It was used the Vitringá method (Ingámed, Paraná) for vitrification. The occurrence of pregnancy per transfer was 66.6% (8/12) and the cumulative pregnancy rate, up to 3 transfers, was 100% (8/8). Only one case was biochemical pregnancy.

**COMMENTS:** Traditionally, the achievement of fresh embryo transfer has been proposed in all FIV cycles, except in special cases, as OHSS risk, inappropriate endometrial preparation and supernumerary embryos. In this case series study, the vitrification technique was able to maintain de embryo viability, obtaining satisfactory outcomes after frozen-thawed embryo transfer. One might question the possibility of implementing in clinical practice the elective vitrification of all embryos in VIF cycles, and postpone embryo transfer for a second moment, with an adequate endometrial thickness, in order to improve endometrial receptivity.

## P-26. Comparative Study of Assisted Reproduction Results in Patients Submitted to Alternative Methods of Attainment Spermatozoa

**M. Santos^1^, V. Cavalcanti^1^, M. Araujo^1^, A.P. dos Reis^1^, C.P. Barbosa^1^**

*^1^Center of Human Reproduction and Genetics - Department of Human Reproduction - Faculdade de Medicina do ABC, Santo André /SP, Brazil*

**OBJECTIVE:** To evaluate normal fertilization rate, normal embryonic cleavage and number of embryos on the third day of development compared to the control group.

**MATERIALS AND METHODS:** Case-control study, 20 patients with Non- Obstructive Azoospermia (NOA), mean age 45.1(± 22.6) and 20 patients with Obstructive Azoospermia (OA) post-vasectomy as a control group, mean age 38.6(± 8:53). The spermatozoa were obtained through alternative methods: PESA (Percutaneous Epididymal Sperm Aspiration) and TESE (Testicular Sperm Extraction), all proceeding were performed for fertilization by ICSI (Intracytoplasmic Sperm Injection). All patients with genetic alterations or female infertility factors were excluded from the study. For procedure of PESA was used connected 1ml syringe, the liquid withdrawn from the epididymal was added to the Petri dish and visualized using an inverted microscope, the samples were washed with buffered culture medium for the procedure and remained at room temperature until the time of ICSI. TESE procedure was performed for testicular biopsy. Testicular fragments were removed and added to the buffered culture medium. Fragments were dissected and observed on an inverted microscope and maintained at room temperature until the moment of ICSI. Data were statistically analyzed with SPSS13.0 software for Windows and used the chi-square test, and considered statistically significant values with *P*<0.05.

**RESULTS:** Comparison of normal fertilization rate and embryo number on the third day of development between the two groups showed no statistically significant difference, resulting in a *P*=0.94 and *P*=0.10 respectively. The normal rate of embryonic cleavage compared among groups showed a statistically significant difference for the control group (*P*=0.02).

**CONCLUSIONS:** Normal embryonic cleavage in control group showed a statistically significant difference when compared to case group (*P*=0.02). Regarding the rate of normal fertilization and embryo number on the third day of development, there was no statistically significant difference between groups. It is believed that with the increase of the study participants, it is possible to find statistical association between variables.

## P-27. The Influence of Immunotherapy with Paternal Lymphocytes in the Results of *In Vitro*Fertilization In Couples With Implantation Failure

**I.N. Machado^1,2^, Y. Klimesch^1,2^, R. Barini^1,2^**

*^1^Allovita Clinical Reproductive Immunology Laboratory, Campinas - SP, Brazil*

*^2^University of Campinas (FCM - UNICAMP), Campinas - SP, Brazil*

**OBJECTIVE:** To present the results of a group of couples with at least 2 cycles of in vitro fertilization and embryo transfer (IVF & ET) without success in achieving pregnancy after being submitted Immunotherapy with Paternal Lymphocytes (IPL).

**MATERIAL AND METHODS:** Retrospective descriptive study conducted from January 2007 to July 2012, which included couples with ≥ 2 cycles of IVF & ET with good quality embryos and serum beta-hCG negative. These couples underwent IPL (≥ 2 doses) and showed positivity Crossmatch (by flow cytometry) before retry IVF & ET. Pregnancy rate (positive beta-hCG and / or visualization of the embryo on ultrasound) and their evolution were evaluated.

**RESULTS:** 53 couples were included, with mean age of 35.7 years (26-48) for women and 38.2 years (28-58) for men. The number of prior IVF & ET ranged 2-9 (average 2.9). The pregnancy rate was 66% (35/53). Of the total of 35 who became pregnant, 2 evolved to abortion and 1 ectopic pregnancy. The success rate found was 60.4% (35-3/53). Of 32 pregnancies that progressed, 5 were twins and 1 triplet. The mean gestational age at delivery was 35.5 weeks (24-39 weeks), with a prevalence of 23 C-sections (72%). The rate of term deliveries (≥ 37 weeks) was 53.1% (17 deliveries) and between 32 and 36 weeks were 12 births (37.5%). Of the 3 births below 32 weeks (9.4%), 1 was a twin at 30 weeks, 1 triplet at 30 weeks and 1 was an abruptio placentae (AP) at 24 weeks. The total number of live births was 38, with a rate of live births 88.6% (31/35 couples) and 1 stillbirth (singleton pregnancy, at 24 weeks, AP).

**CONCLUSIONS:** The pregnancy rate and live birth deliveries found for the studied group showed that this group seems to benefit from IPL, suggesting that IPL can be considered a valuable option of adjunctive therapy in cases of implantation failure in IVF & ET cycles.

## P-28. The Effect of Mesenchymal Cells in Retrocervical Endometriosis in an Experimental Model

**J.P. de Abreu^1^, T.F. do Amaral^1^, C.A. Savari^1^, C.L.K. Rebelatto^1^, L.G.A. Capriglione^1^, L. Miyague^1^, L. de Noronha^1^, V. F. do Amaral^1,2^**

*^1^Programa de Pós-graduação em Ciências da Saúde da Pontificia Universidade Católica do Paraná - PUCPR*

*^2^Universidade Federal do Paraná - UFPR*

**OBJECTIVE:** Endometriosis is a chronic disease characterized by the presence of ectopic endometrial tissue. This disease is common and affects 5-15 % of women in the reproductive age. Endometriosis in a deeply way can afecct up to 20% of their carriers. Among its main clinical manifestations are dysmenorrhea, dyspareunia, and infertility. Despite its high morbidity, the pathogenesis of endometriosis is still not entirely clear. Currently, research groups try to find a correlation between endometriosis and stem cells, among which mesenchymal cells. Studies have already demonstrated the presence of stem cells in animal and human endometrium; in the same way as research lines attempting to show that epithelial-mesenchymal transition that occurs in the human endometrium has similarity to the pathophysiology of endometriosis. In this paper, we propose to observe the effect of mesenchymal cells in endometriosis implants created in rabbits .

**MATERIAL AND METHODS:** To this end, 27 New Zealand rabbits which were divided in three groups were used: Endometriosis group, where the implant retrocervical endometriosis was performed, Mesenchymal group, that in addition to the creation of disease, mesenchymal cells were applied and the Control group, designed to remove the bias manipulation of the uterus of animals. Were analyzed the fertility rate, the time taken to enter the fertile period, obstetric outcomes, the difference in the size of the implants and the histology of the same.

**RESULTS:** We obtained as fertility rate the following values: 77.78 % for the Control group and the Endometriosis group and 11.2 % for Mesenchymal group (*P*= 0.015). Regarding the time to start the fertile period, the Mesenchymal group was what else was late, with P=0.005. Analyzing the obstetric outcomes, we found that the Control group showed the seven pregnancies, two stillbirths, three miscarriages and two live births; Endometriosis group already had two stillbirths and five live births. The mesenchymal group had a low fertility rate and lacked further analysis. The difference in the sizes of implants (final - initial) was characterized by the growth in Mesenchymal groups, but no statistically significance (*P*=0.83). About the macroscopic appearance of the lesions, Mesenchymal group showed the greatest adhesion, and about the histology of the implants, the Mesenchymal group had one animal more than Endometriosis group wich Keenan Index 3 (well characterized endometrium), although this not being statistically significant (*P*=0.730).

**CONCLUSIONS:** We conclude that applying mesenchymal cells in endometriosis implants of rabbits we harmed the fertility rate, we delay the entry in the fertile period and harmed both the histology and the anatomy of the lesion.

## P-29. Endometrial Injury in Women Undergoing Assisted Reproduction Techniques

**C.O. Nastri^1^, R.A. Ferriani^1^, W.P. Martins^1^**

*^1^Setor de Reprodução Humana, Departamento de Ginecologia e Obstetrícia, Faculdade de Medicina de Ribeirão Preto, Universidade de São Paulo*

**OBJECTIVE:** To identify, appraise and summarize the existent evidence regarding the effect of endometrial injury performed between day 7 of the previous menstrual cycle and day 7 of embryo transfer cycle in women undergoing assisted reproductive techniques.

**MATERIAL AND METHODS:** A systematic review and meta-analysis of randomized clinical trials (RCTs) that evaluated the endometrial injury in the period of interest versus no intervention or sham procedure. The search was conducted in 13 databases. The data were analyzed according to the intent-to-treat principle. The quality of the studies was assessed by examining the risk of selection, attrition, performance, detection bias, selective reporting and other sources of bias; a funnel plot was used to examine the risk of publication bias. Data synthesis for clinical pregnancy and live birth was performed using relative risk (RR), and its precision was examined by the 95% confidence interval (CI). The inconsistency across studies was evaluated subjectively and by I^2^. The quality of evidence would be downgraded if the results were obtained from poor quality studies, if the estimates were imprecise, if the results were inconsistent across studies, or if we suspect from publication bias.

**RESULTS:** The search was conducted in March-2014. Ten studies were included. Two studies were considered to have high risk of bias and not suspect publication bias. Nine studies performed endometrial injury in the luteal phase of the previous cycle and one study on the third day of the same cycle of embryo transfer. There was a significant increase in clinical pregnancy (RR=1.41; 95% CI = 1.19-1.67; 10 RCTs; 1,426 women, I^2^=21%, highquality evidence) and live birth rates (RR = 1.43; 95% CI = 1.19-1.71; 6 RCTs; 950 women, I^2^=0%, high-quality evidence).

**CONCLUSIONS:** There is high quality evidence showing that endometrial injury performed in this period improves the chance of clinical pregnancy and live birth. However, one should consider that most of the included women had not achieved pregnancy after multiple prior embryo transfers and the results of this review should not be generalized to all women.

## P-30. Cryopreservation of Supernumerary Zygotes at the Two Pronuclear Stage Allows an Increase of Cumulated Pregnancies

**A. Senn^1^, F. Urner^1^, A. Chanson^1^, F. Murisier^1^, D. Wirthner^1^, M. Megalo^1^, M.Germond^1^**

*^1^Centre Hospitalier Universitaire Vaudois (CHUV), Centre de Procréation Médicalement Assistée (CPMA), Lausanne, Switzerland*

**OBJECTIVE:** The Swiss law on IVF prohibits cryopreservation of embryos after syngamy, but allows freezing at the pronuclear stage. Systematic cryopreservation of all supernumerary zygotes has been performed since 1993. The purpose of this study is to report on the cumulated pregnancy rates after transfer of fresh and cryopreserved zygotes in ICSI cycles over 18 years (1993-2010).

**MATERIAL AND METHODS:** All patients enrolled in an ICSI cycle, stimulated with recombinant-FSH (Gonal-F, Merck-Serono; Puregon, Organon) under a long agonist protocol (Decapeptyl, Ferring) were selected retrospectively. A maximum of 2-3 zygotes were left in culture and the remaining zygotes immediately frozen (slow protocol). Transfers occurred on day 2-3, exceptionally on day 5. In the absence of pregnancy, 2-3 cryopreserved zygotes were thawed and transferred. The procedure was repeated until a pregnancy occurred or until all cryopreserved zygotes were thawed. A total of 2’836 fresh cycles and 4’318 corresponding frozen-thaw cycles were analysed. The fresh cycles were classified in 6 groups according to the number of recovered oocytes (1-4; 5-9; 10-14; 14-19; ≥20). For each of these groups, the implantation rates in fresh and cryo transfers and the cumulated conception rates were calculated.

**RESULTS:** In three age categories (A:<35, B:35-38, C:>38 years), fresh clinical pregnancies were independent on the number of oocytes recovered (*P*>0.5). The cumulated pregnancy rates augmented linearly (*P*<0.001) from 32.4% to 82.1%, from 22.5% to 89.9%, and from 13.0% to 64.1 % for the age categories A, B and C respectively as the number of recovered oocytes varied from 1-4 to ≥20 oocytes.

**CONCLUSIONS:** Egg quality and implantation rates of embryos derived both fresh or frozen-thawed zygotes are independent on the number of oocytes retrieved in cycles using long stimulation protocols and ICSI. In conditions where no embryo selection or embryo freezing can be performed, cryopreserving fertilized oocytes at the 2 pronuclear stage allows achieving high and consistent pregnancy rates by taking advantage of the pregnancy potential of all recovered oocytes.

## P-31. Ovarian Hyperstimulation Syndrome with an Evolution to a Clinical Status of Thrombotic Microangiopathy

**H.J.P. Cardim, C.B. Dartibale, A.P. Bonfim, G.C. de Freitas, S. Pagliarini e Silva, R. Oyama**

*Universidade Estadual de Maringá/PR*

*FertClínica - Maringá/PR*

**INTRODUCTION:** The ovarian hyperstimulation syndrome (OHSS) is a marked response to ovarian stimulation therapy, triggered by the administration of human chorionic gonadotropin (hCG), which induces the overproduction of vascular endothelial growth factor, resulting in a widespread increase in capillary permeability.

**CASE REPORT:** S.J.M.T., 32 years old, Caucasian. Sought treatment at the fertility clinic with the desire to become pregnant and indication of in vitro fertilization (IVF). The patient was subjected to the ovarian stimulation with high response to the gonadotropin and risk of hyperstimulation, analog of gonadotropin releasing hormone it was administered instead of hCG. The patient’s serum estradiol was 10.000pg/ml and developed abdominal pain in both iliac fossa associated with nausea, the day after aspiration. The initial transvaginal ultrasound showed right and left ovary with a volume of 123cm^3^ and 107.3cm^3^, respectively, and a moderate amount of fluid in the pouch of Douglas. The embryo transfer was canceled and the patient was treated with cabergoline and albumin. After 8 days, the patient developed oliguria and clinical and laboratory worsen with thrombocytopenia (16.000/ mm3), anemia (7.4g/dL), lactate dehydrogenase (1.195U/L), total bilirubin (1.78), and creatinine (3.34mg/ dL), and was handled with dialysis and plasmapheresis. On the ninth day, the patient had severe respiratory failure. The investigation of rheumatologic tests were negative. On the fifteenth day there was clinical and laboratory improvement, except creatinine which remained elevated for 1 month.

**COMMENTS:** The patient submitted to ovarian stimulation developed a clinical status of thrombotic microangiopathy (hemolytic anemia, thrombocytopenia and renal failure), making differential diagnosis between thrombotic thrombocytopenic purpura (TTP) and hemolytic uremic syndrome (HUS).The good outcome after plasmapheresis agrees with the hypothesis of TTP. However, the evolution to severe renal failure reinforced the possibility of HUS. There are many causes of atypical HUS, it is known that there is an association of these syndromes with hyperestrogenic states such as pregnancy, puerperium and oral contraceptive use; but there is no description in the literature of the association of IVF, OHSS and HUS. So, it validates the case description above that demonstrates the association of ovarian hyperstimulation and HUS, being a likely factor associated hyperestrogenism.

## P-32. Does the Serum Concentration of Vitamin D Impact in Endometrial Thickness in IVF Cycles?

**V.M. Lopes, J.P.B. Brasileiro, M. Roller, L. Noleto, E.F. Duarte, J.R.C. Lopes**

*Instituto Verhum - Brasília*

*Cenafert - Salvador*

**OBJECTIVE:** To evaluate the relationship between endometrial thickness and serum concentration of vitamin D in patients undergoing IVF/ICSI.

**MATERIAL AND METHODS:** This is a multicenter transversal study. All patients submitted to controlled ovarian hyperstimulation (OHS) for oocytes retrieval between November 2013 and May 2014 who accepted to participate were included in the analyses; this study was approved by the ethics committees from the involved institutions. A venous blood sample for the determination of serum concentration of vitamin D was taken at the time of oocytes retrieval. The endometrial thickness was measured by ultrasound on the same day of trigger injection. The variables analyzed included: age, body mass index (BMI), OHS protocol (GnRH agonist or GnRH antagonist) and serum concentration of vitamin D (<20; 20.0-29.9 e ≥30 ng/ml). Uni and multivariate analyses (by logistic regression) were used for estimating the crude and adjusted predictive value of variables of interest in relation to endometrial thickness; these estimates were described as odds ratios and their respective 95% confidence intervals (CI 95%).

**RESULTS:** A total of 241 cases de OHS were analyzed. The mean endometrial thickness was 10.2 mm (CI 95%:9.510.9), 10.4 mm (CI 95%:9.8-10.9) and 9.7 mm (CI 95%:9.1-10.2), respectively, for levels of vitamin D of <20 (N=26), 20.0-29.9 (N=92) and ≥30 ng/ml (N=75). The mean age and BMI were 35.6 years (CI 95%:33.8-37.4) e 23.8 (CI 95%:22.7-24.9); 36.2 years (CI 95%:35.2-37.2) e 23.9 (CI 95%:23.2-24.6); e 35.6 years (CI 95%:34.536.6); e 23.2 (CI 95%:22.5-24.0), respectively, for levels of vitamin D of <20, 20.0-29.9 and ≥30 ng/ml. For 164 cases assigned to GnRH antagonist OHS protocol, the mean serum vitamin D concentration was 28.7 ng/ml (CI 95%: 27.0-30.4). For 58 patients assigned to GnRH agonist OHS protocol, the mean serum vitamin D concentration was 29.1 ng/ml (CI 95%:27.0-31.2). No association was found between endometrial thickness and serum vitamin D concentration (OR=0.40; CI 95%:0.10-1.48), even after adjustment for age, OHS protocol and BMI in multivariate analyses (OR=0.39; CI 95%:0.12-1.22).

**CONCLUSIONS:** In patients undergoing IVF, there is no association between endometrial thickness and serum vitamin D concentration, independent of age, OHS protocol and BMI.

## P-33. AMH: Marker of Embryo Quality

**E. De Conto^1^, D.S. da Silva^1^, V.K. Genro^1^, R.Chapon^1^, J.S.L. da Cunha Filho^1^**

*^1^Centro de Reprodução Humana Insemine*

**OBJECTIVE:** To determine whether there is influence of serum anti-Müllerian hormone (AMH) on embryo quality in assisted reproduction cycles.

**MATERIAL AND METHODS:** Prospective cohort study in which 248 patients were allocated with infertility of unknown cause (13 cases), endometriosis (102 cases), tubal factor (71 cases) and male factor (62 cases) between January 2011 and May 2014. Induction protocol used was the same for all patients and samples collected for analysis of serum AMH and FSH were carried out on the third day of the cycle. The embryo score (score 0-100) is based on early cleavage, cell division and fragmentation. Statistical analysis was performed linear regression to compare the embryo scoring with patient age, serum FSH, serum AMH levels and causes of infertility (SPSS version 20.0).

**RESULTS:** The result of linear regression showed that serum AMH (*P*=0.001, 95% CI = 5.71; 21.53) and FSH (*P*=0.024, 95% CI =-13.35;-0,96) levels are related to embryo quality, and there is greater influence of AMH. The patient’s age (*P*=0.435) and the cause of infertility (*P*=0.231) had no effect on the quality of the embryos obtained in this cohort.

**CONCLUSIONS:** It has been shown that AMH is a sensitive marker of ovarian and prognostic factor chance of pregnancy and birth reservation. We demonstrate for the first time that the level of AMH is related too with the quality of the embryos obtained by in vitro fertilization, can be used as a marker to direct the conduct in assisted reproduction treatments so as to obtain better quality embryos.

## P-34. Hemoperitonium After Follicular Aspiration Guided by Ultrasonography - Series of Cases

**É.V.S. Jordão^1^, A.A. Silva^1^, A.C.P. Barbosa^1^, B.R. de Carvalho^1^, F.S. Estrela^1^, H. Nakagawa^1^**

*^1^Clínica GENESIS Brasilia/DF*

**INTRODUCTION:** Currently, follicular aspiration guided by transvaginal ultrasonography (TVS) is the method of choice for oocyte retrieval in assisted reproductive technology cycles. Although safe, it is not without complications, among these the hemoperitoneum. We report three cases of hemoperitoneum, in which patients were treated surgically.

**CASE REPORT:** Case 1: 23 years old, male factor infertility, subjected to follicular aspiration guided by TVS, with uptake of 26 oocytes. Showed clinical and laboratory signs of acute anemia and underwent laparotomy 12 hours after the puncture. Bleeding was identified at the hilum, controlled with hemostatic sutures. Case 2: 32 yers old, male factor infertility, subjected to follicular aspiration guided by TVS, with uptake of 9 oocytes. Presented abdominal pain and distention about 15 hours after the puncture, with hemodynamic and hematology consequences and moderate amount of free fluid in the cavity. Underwent laparotomy and found hemorrhagic corpus luteum, held with oophoroplasty. Developed pulmonary edema, acute, intensive care is needed. Case 3: 41 years old, ovarian and tubal factor infertility, subjected to follicular aspiration guided by TVS, with uptake of 5 oocytes. Presented with abdominal pain and distension with hemodynamic and hematological changes and moderate amount of free fluid in the cavity found about 12 hours after the puncture. Underwent laparotomy for hemostasis with oophoroplasty of ruptured corpus luteum.

The three patients were screened for coagulopathy, needed blood transfusions and underwent embryo freezing.

**COMMENTS:** Although rare, hemoperitoneum after follicular aspiration guided by TVS is a serious complication, benefiting the patient for early diagnosis and appropriate approach. Is attributed mainly to inadvertent puncture of ovarian, vaginal or iliac vessels, or, more rarely, coagulopathies previously unknown.

In our center, representing 0.1% of the IVF / ICSI effect similar to that in the literature, ranging between 0.06% and 0.2%. In order to minimize the risks of follicular aspiration guided by TVS, it is recommended: attention to personal and / or family history of clotting disorders, being able to adopt the tracking pretreatment coagulopathy; sequential follicular aspiration with single puncture of the vaginal wall; and recovery of signs and / or symptoms of hemodynamic instability in the following oocyte retrieval hours.

## P-35. *In Vitro* Fertilization of Mice Using Different Commercial Media

**I.S. Chaussard, V.L.L. Amaral, M Frajblat, A.Senn**

*UNIVALI- Universidade do Vale do Itajaí - Itajaí, Santa Catarina;*

*UFRJ - Universidade Federal do Rio de Janeiro - Rio de Janeiro, Rio de Janeiro*

*FABER - Foundation pour’l Andrologie, la Biologie et l’Endrocrinologie de la Reproduction - Lausanne, Switzerland*

**OBJECTIVE:** In vitro fertilization (IVF) of mice has been extensively used for different purposes, among them studies associated to the physiology of the gametes, in the production of different strains of mice and as an important tool for the development of biotechnologies in the Assisted Human Reproduction (AHR). Many media for fertilization have been developed in order to promote the success of fertilization.

The objective of this study was to evaluate the efficiency of different commercial media for fertilization and embryo culture routinely used in the AHR, during IVF mice.

**MATERIAL AND METHODS:** B6D2F1 mice (DBA/2J x C57Bl/6J) were used in this study. Females were intraperitoneally stimulated with 10 IU of equine chorionic gonadotropin (Novormon - Schering-Plough^®^), after 48 hours 10 IU human chorionic gonadotropin (Vetecor - Hertape Calier^®^) was administered.

After 17 hours, the oocytes were collected in GV-Hepes medium (Ingaméd^®^), denuded in 50 µL of hyaluronidase (Ingáse®, Ingaméd®) during 30 seconds, then, selected and placed with capacitated spermatozoa. Fertilization was carried on IVF (Vitrolife^®^), Global (Global Life^®^) Single Step Medium (Irvine^®^) and GV-FERT (Ingaméd^®^) media, all supplemented with 10% of synthetic serum (Ingaméd^®^) in volume of 300µL per well.

After 24 hours, the fertilization rates were assessed by the number of 2 cells embryos and the results were analyzed using the chi-square test and the differences were considered significant when *P* <0.05.

**RESULTS:** The media IVF (Vitrolife^®^), indicated for fertilization, and Single Step Medium (Irvine®), indicated for embryo culture, promoted higher fertilization rates (44.3% and 38.1%) without statistical difference between them.

The media GV-FERT (Ingaméd^®^) and Global (Global Life^®^) showed fertilization rates of 21.5% and 12.2% respectively.

**CONCLUSIONS:** The results showed that fertilization and embryo culture commercial media, IVF (Vitrolife^®^) and Single Step Medium (Irvine^®^), have the same efficiency to promote fertilization of murine oocytes.

The media GV-FERT (Ingaméd^®^) and Global (Global Life^®^) were less efficient, however, also have the ability to support fertilization. Despite having different efficiency, both fertilization and culture media can be applied to mice IVF.

## P-36. GnRH Agonist Versus GnRH Antagonist for *In Vitro* Fertilization/Embryo Transfer Cycles, in Cases of Good Prognosis

**R.B. de Morais^1^, A.C.P. Barbosa^1^, I.O. Cabral^1^, H.M. Nakagawa^1^, A.A. Silva^1^, B.R. de Carvalho^1^**

*^1^GENESIS - Centro de Assistência em Reprodução Humana, Brasília, Distrito Federal*

**OBJECTIVE:** To compare results of protocols using GnRH agonist (aGnRH) and GnRH antagonist (antGnRH), in cycles of in vitro fertilization with intracytoplasmic sperm injection and embryo transfer (ICSI/ET), in patients considered of good prognosis.

**MATERIAL AND METHODS:** Retrospective analysis of 650 ICSI/ET cycles performed between January 2008 and December 2012, in a private center for assistance on human reproduction. Inclusion criteria were: cycles performed with long aGnRH protocol or with antGnRH protocol; oocytes fertilized with sperm from partner in freshly obtained semen; and embryos transferred in the treatment cycle. We excluded intrauterine insemination cycles converted to ICSI (n = 41) or PGD (n = 5), women older than 40 years (n = 144) and oocyte donors (n = 26). The final sample was 434 cycles, divided into two groups: aGnRH (n = 291) and antGnRH (n = 143). Pre-gestational, gestational and perinatal outcomes were evaluated, considering statistically significant *P*< 0.05.

**RESULTS:** There were no significant differences between the groups, considering patients’ average age, duration of ovarian stimulation and number of dominant follicles. Patients in aGnRH group presented significantly higher amounts of total oocytes (aGnRH: 10.7 ± 5.7 vs antGnRH: 9.3 ± 5.7, *P*<0.01), mature oocytes (aGnRH: 8.2 ± 4, 5 vs antGnRH: 6.8 ± 4.4, *P*<0.001) and good quality embryos transferred (aGnRH: 1.7 ± 0.8 vs antGnRH: 1.5 ± 0.9, *P*<0.05). Fertilization and embryo implantation rates were higher for aGnRH group (72.8% [1612/2214] and 27.6% [178/644]) compared to antGnRH (65.3% [602/922] and 14, 7% [43/293], *P*<0.0001).

There was no difference between rates of pregnancy loss and, thus, rates of live births were also higher for the first group (aGnRH: 48.8% [142/291] vs antGnRH: 26.6% [38/143]; *P*<0.0001).

There were no significant differences between rates of preterm delivery or birth weight, comparing the two groups.

**CONCLUSIONS:** In ICSI/ET cycles of patients considered to have a good prognosis, the long GnRH agonist protocol seems to be still the one offering the best reproductive outcomes.

## P-37. Reproduction PostMortem: How the Brazilian Society has Morally Assimilated this Possibility?

**M.C.B. de Souza^1^, A.L.S.R. Costa^1^, A.C.A. Mancebo^1^, R.A.A Antunes^1^, M.M. de Souza^1^, T.R. Panaino^1^**

*^1^FERTIPRAXIS Rio de Janeiro/RJ*

**OBJECTIVE:** The Civil Code of 2002, concerning fatherhood, included in the legal presumption of parentage hypotheses related to assisted reproduction. The Federal Council of Medicine -CFM Resolution 2013/2013, though without the force of law, sets ethical standards for the use of ART, considering among others, the need to harmonize the use of these with the principles of Ethics. The speed of scientific advances in science, particularly in the field of reproduction, contrasts with the slowness of law to accompany such a process. How are users considering the possibility of post-mortem reproduction?

**MATERIAL AND METHODS:** One year after the new CFM resolution, we proceeded survey of IC completed by users of ART in the Service in the 2nd semester of 2013 and first quarter of 2014. We checked the possibility of using embryos, oocytes or sperm post-mortem.

**RESULTS:** There were 236 procedures performed, 190 IVF / ICSI cycles (173 with own gametes, 08 recipients of donor eggs and 09 donants), 27 women undergoing freezing of oocytes and 19 semen cryopreservation. In IVF / ICSI cycles 133 (70%) authorize the use of embryos postmortem. The decision-making power over the embryos rests with the surviving member in 128 cases (96%), with an option on 03 cases for donation to research and 02 to other couples. In the event of death of the couple, opinions are divided: 26%(35) opt for donation to research, 43% (57) donate to other couples at the discretion of the clinic and 31% (41) define disposal. As for frozen eggs, 23 out of 27 women request the disposal in case of death (04 donates to another patient at the discretion of the clinic) while the 19 men who cryopreserved semen, 63% (12) authorize the use of by the partner and others want prompt disposal.

**CONCLUSIONS:** Users of ART today manifest in favor to the possibility of a pregnancy in the absence of one of the spouses, as well as the donation of embryos or even the plain and simple disposal in the absence of both. Evidence is open to new reflections and discussions, both from the affective point of view and of Law.

## P-38. Impact of Severe Male Factor in Genetic Screening Preimplantation

**M. Riboldi^1,2^, L. Rodrigo^2^, C. Rubio^2^, N. Al-Asmar Piñar^3^, B. Coprerski^1^, C. Simon^2,4^**

*^1^IGENOMIX Brasil, Laboratório de Medicina Genética, São Paulo, Brasil*

*^2^IGENOMIX Espanha S. L.. Parque Científico, Valencia, Espanha*

*^3^IGENOMIX USA, Miami, Estados Unidos ^4^Departamento de Obstetrícia e Ginecologia da Universidade de Valencia, Valencia - Espanha*

**OBJECTIVE:** Evaluate the rate of embryonic chromosomal abnormalities through Pre- implantation Genetic Screening (PGS) in couples with reproductive age and the presence of severe male factor.

**MATERIAL AND METHODS:** From 2011 to 2013, couples who sought assisted reproduction treatment with reproductive age (<38 years) with no history of implantation failure (RIF), recurrent miscarriage (RM) and the presence of male factor (MF) as cause of infertility were pre-selected for this study. Patients who had the semen concentration of ≤5 million sperm/mL were targeted directly to the achievement of PGS (embryo biopsy on day 3 of embryonic development and genetic analysis of 24 pairs of chromosomes by CGH-array) and selected for this study.

**RESULTS:** The 66 selected couples had an average sperm concentration of 1.6 million sperm cells/mL. The average number of mature oocytes (MII) and embryos analyzed were 12.2 and 6.9, respectively. The results allowed the analysis of 458 embryos with 69.7% abnormal embryos (320), 15.5% (71) chaotic anomalies, 5.6% (26) partial aneuploidies and 8.1% (37) had ≤ 3 anomalies. The screening of the 24 chromosomes allowed the diagnosis of 87 (18.9%) embryos, which by FISH technique would not have been detected as abnormal and thus prevented their transfer. These analyzes allowed an average of 1.5 embryos transferred to 58 couples, resulting in a rate of 87.9% of transfer. The pregnancy rate/transfer were 72.4%, pregnancy/ cycle 63.6%, implantation of 62.8% (54/83) and abortion rate 2.4% (1).

**CONCLUSIONS:** The analysis by PGS showed high percentages of chromosomal abnormalities in patients with severe male factor, enabled the transfer of chromosomally normal embryos and achieved high rates of pregnancy and lower miscarriage.

## P-39. Can Higher Normal Sperm Count, Compared with Lower Normal Sperm Count, Improve IVF/ICSI Results? A Case Control Study

**M.S. Approbato^1^, M.S. Ramos^1^, M.C.S. Maia^1^, T.M. Silva^1^, S.R.C. Rodrigues^1^, M.Z. Brito^1^**

*^1^Laboratório de Reprodução Humana, Hospital das Clínicas, Universidade Federal de Goiás*

**OBJECTIVE:** To evaluate if 40 x 106 or more normal sperm count improves IVF/ICSI results when compared with 20 to 39 x 106 sperm count (lower normal).

**MATERIAL AND METHODS:** Case-Control study with paired confound factors. We compared 30 IVF/ICSI patients with 40 x 10^6^ sptz/ml or more normal sperm count (WHO, 1999) with 30 IVF/ICSI patients with 20 to 39 x 106 sptz/ml sperm count (lower normal, controls). The following confound variables were paired: Age, Infertility time (Years), Sperm morphology (% normal). Excluded cases with < 20 % normal morphology), FSH (excluded cases with FSH >= 10 mUI/ml). Main outcome measures: Fertilization rate, Embryo cleavage, Chemical pregnancy (Positive b-hCG). Statistical tests: Mann-Whitney and Chi-Square. P < 0,05.

**RESULTS:** There were no statistical differences for all paired variables. We did not find statistical differences in fertilization rate, embryo cleavage, and a chemical pregnancy (Positive b-hCG), between the groups.

**CONCLUSIONS:** When compared to 20 to 39 x 106 sptz/ ml sperm count, semen concentration >= 40 x 106 did not improve IVF/ICSI fertilization rate. Semen concentration >= 40 x 106 did not improve IVF/ICSI embryo formation rate. Semen concentration >= 40 x 106 did not improve IVF/ICSI pregnancy rate (beta hCG+).

## P-40. Systemic and Follicular Oxidative Stress in Infertile Women with Minimal/Mild Endometriosis Undergoing Controlled Ovarian Stimulation for ICSI: Is There a Role in the Etiopathogenesis of Infertility?

**M.G. Da Broi^1^, J.K. Rodrigues^1^, A.Z. Andrade^1^, V. Giorgi^1^, A.A. Jordão^1^, P. Navarro^1^**

*^1^Faculdade de Medicina de Ribeirão Preto (FMRP) - USP*

**OBJECTIVE:** The mechanisms involved in the pathogenesis of endometriosis-related infertility, especially in early stage pelvic disease, have not been fully elucidated. The oxidative stress (OS) role in this condition has been questioned. No study to date has evaluated jointly different antioxidant and pro oxidant markers in serum and follicular fluid (FF) of women with infertility related to minimal/mild endometriosis (EI/II). The aim of this study was to compare eight OS markers in serum and FF of infertile women with and without EI/II undergoing controlled ovarian stimulation (COS) for intracytoplasmic sperm injection (ICSI).

**MATERIALS AND METHODS:** From October 2009 to October 2010, 69 serum samples (25 with EI/II and 44 without EI/II - with male or tubal factor of infertility) and 51 FF samples (19 with EI/II and 32 without EI/II) were collected in the day of oocyte retrieval and had data analyzed. Total hydroperoxides (FOX1), malondialdehyde (MDA), advanced oxidation protein products (AOPP), glutathione (GSH), superoxide dismutase (SOD) and the total antioxidant capacity (TAC) were determined by spectrophotometry, vitamin E (Vit E) by high performance liquid chromatography, and 8-hydroxy-2’ -deoxyguanosine (8OHdG) by ELISA. Total protein (pt) levels were determined by Labtest Kits. Significance level was set on 5%.

**RESULTS:** We observed higher serum concentrations of FOX1 (8.48±1.72 µmol/g pt) and lower serum concentrations of TAC (0.38±0.18 mEq Trolox/L), and higher follicular concentrations of 8OHdG (24.21±8.56 ng/mL) in infertile women with EI/II compared to those without EI/II (7.69±1.71 µmol/g pt, 0.46±0.15 mEq Trolox/L, 17.22±5.6 ng/mL, respectively).

**CONCLUSIONS:** We evidenced the occurrence of systemic and follicular OS in infertile patients with minor endometriosis undergoing COS for ICSI. For the first time it was demonstrated the presence of higher follicular concentrations 8OHdG in women with EI/II, a marker of DNA oxidative damage, which might be related to compromised oocyte quality. Thus, we suggest that the OS may be involved in the etiopathogenesis of minor endometriosis -related infertility.

**Support:** CNPq, FAPESP (2008/58197-6), Brazil.

## P-41.Testicular Sperm Extraction (Tese) in “Sertoli Cell Only” Syndrome

**P.F. Taitson^1^, A. Mourthé Filho^1^**

*^1^Discipline of Human Reproduction and discipline of Medical Clinics (Urology) - ICBS/PUC Minas*

**OBJECTIVE:** The syndrome of “Sertoli cell only” is an unusual finding, with an incidence of emphasis between nonobstructive azoospermia. The diagnosis is determined by testicular biopsy and detailed pathological study. His description is restricted to cases where biopsies of testicular Sertoli cells exhibit only. The tubules are filled by narrow sertoli cells appearance, often uniform. Its nucleus is more elongated than usual, but they are nearly usually lobed. Condensation of chromatin and nucleoli changed sizes are displayed. As there is an absence of germ cells, the apical cytoplasm of adjacent cells showing interdigitations intense. The objective of this study was to investigate the literature, the presence of spermatozoa in patients undergoing testicular biopsy diagnosis of “Sertoli cells only” syndrome.

**MATERIAL AND METHODS:** We searched the databases of Medline from 2003 to 2013 articles with the following keywords: TESE, Sertoli cell-only syndrome, nonobstructive azoospermia.

**RESULTS:** 130 articles, which, after treatment with meta -analysis showed sperm in 41% (± 3.2) were found. The parameters analyzed: nonobstructive azoospermic patients undergoing TESE and diagnosed with Sertoli cell only syndrome.

**CONCLUSIONS:** The authors conclude that the technique TESE targeting the recruitment of gametes, should be guided by exhaustion in the evaluation of the fragment obtained, because even with the definition of the table above, it is possible to find cells of spermatogenic lineage in a considerable proportion of individuals as literature has highlighted.

## P-42. Monozygotic Twins in Assisted Reproduction: Laser Assisted Hatching as a Risk Factor

**K.H. Ribeiro^1^, V.N. Perez^1^, M.H. Ribeiro^1^, G.C. Silva^1^**

*^1^CLINIFERT - Centro de Reprodução Humana / Florianópolis - SC*

**OBJECTIVE:** To evaluate the impact of Laser Assisted Hatching (AH) in rates of monozygotic pregnancy and multiple pregnancy in ICSI cycles in various age groups.

**MATERIAL AND METHODS:** Retrospective analysis of 765 ICSI cycles conducted between 2008 and 2012, with and without Laser Assisted Hatching, stratified into 4 age subgroups. In the first group, 435 cycles with AH. Included patients with previous ICSI failures, high FSH, increased age and embryos with pellucid zone (ZP)> 16um or increased resistance. Another group of 330 cycles included all other cases with embryos with standard ZP independent of maternal age. We use OCTAX laser system, 2 to 4 pulses in a single point of ZP (10-12 um). Exclusion criteria were frozen and poor quality embryos. Fresh embryos, grade I and II were transferred on day 3 in the AH group and the 3rd to 5th day in the other group. Monozygotic twins (MZT) were identified by ultrasound in 4th and 5th weeks after embryo transfer, using gestational sacs numbers greater than embryos transferred. Used t Student and X2 test with Yates correction for statistical analysis. Main parameters: pregnancy rates, multiple pregnancy and MZT rates.

**RESULTS:** A total of 303 pregnancies, 178 in AH group and 125 in the other group, but no difference in overall pregnancy rates (40.9% vs 37.9%, *P* <0.05.). The overall multiple pregnancy rate was 25.4%, with the highest incidence in the AH group (30.3% vs. 18.4% *P* <0.05), 22.1% was no MZT and 3.3% was MZT pregnancy. The incidence of MZG was significantly higher in the AH group (5% vs. 0.8%, *P* <0.05), with no difference between groups in cases of non monozygotic multiple pregnancy (25.27% vs. 17.6 %, *P* > 0.05). In concerning of the age, it was found high incidence of MZT in AH group in women above 30 years.

**CONCLUSION:** Laser Assisted Hatching is a very important factor in MZT rates in women over 30 years. More studies and considerations about the extension of the laser hole must be used to reduce the incidence of MZT.

## P-43. Triplet Pregnancy in IVF: The Search for Extinction

**V. Lopes, J.P.B. Brasileiro, N.I.T. Zavattiero, M.F. Roller, L. Noleto, J.R.C. Lopes**

*Instituto Verhum - Brasília*

*Cenafert - Salvador*

**OBJECTIVE:** To calculate the 2013 annual rate of multiple pregnancy in patients undergoing in vitro fertilization (IVF) and/or intracytoplasmic sperm injection (ICSI) at an assisted reproduction center and compare it with previous periods.

**MATERIAL AND METHODS:** Descriptive and retrospective study utilizing the institution’s database to calculate the number and percentage of single and multiple pregnancies from January 1^st^ to December 31^st^, 2013. These data were then compared to 2 published statistics for the same center, in 2005 and 2009. All patients undergoing IVF and/or ICSI during the analyzed periods were included in the study. All pregnancies were classified into single, twin, triple or quadruple according to the number of fetuses presenting heart beat in an ultrasound performed at 12 weeks of gestation.

**RESULTS:** As published in 2005, from 351 IVF cycles there were 115 pregnancies (32.3%), being 62 single (53.9%), 36 twin (31.3%), 15 triplet (13%) and 2 quadruplet pregnancies (1.7%). In 2009 the number of cycles was 521 resulting in a total of 220 pregnancies (42.4%) with 123 single (55.9%), 83 twin (37.7%) and 14 triplet pregnancies (6.4%). Data from 2013 showed a total of 291 cycles with 88 resulting ongoing pregnancies (30.2%), being 59 single (67.0%), 28 twins (31.8%) and 1 triplet pregnancy (1.1%). No quadruplet pregnancies were diagnosed in the last 2 periods: 2009 or 2013.

**CONCLUSIONS:** Our statistics show, since 2005, a decrease in the rate of multiple pregnancies, greater than twin, reflecting the practice of transferring a lower number of embryos per procedure. During the same period, the rate of twin pregnancies remained the same. This fact emphasizes the need for identifying high-risk groups and for prioritizing single embryo elective transfers to selected patients.

## P-44. Prediction of HighResponse During Controlled Ovarian Stimulation: Systematic Review and MetaAnalysis

**C.O. Nastri^1^, R. Moroni^1^, V.M. Leitão^1^, D.M Teixeira^1^, W. P. Martins^1^**

*^1^Setor de Reprodução Humana, Departamento de Ginecologia e Obstetrícia, Faculdade de Medicina de Ribeirão Preto, Universidade de São Paulo*

**OBJECTIVE:** Identify, appraise and summarize evidence regarding methods used for prediction of high-response during ovarian controlled stimulation (COS) in women undergoing assisted reproduction.

**MATERIALS AND METHODS:** Systematic review and meta-analysis of cohort studies evaluating methods to predict high-response (≥15 large follicles on hCG day or ≥15 retrieved oocytes) during COS in assisted reproduction. The search was performed in the databases MEDLINE and Scopus. Sensitivity (Se), specificity (Sp), and area under the ROC curve (AUROC) were pooled and expressed with the 95% confidence interval (95%CI). Heterogeneity was evaluated by the I2.

**RESULTS:** Eight studies were included, comprising 5,536 women. The incidence of high-response was 12.0% (CI95% 5.7-20.3%). All evaluated outcomes were determined before the start of COS: antral follicle count (AFC, 6 studies), AMH (8 studies), FSH (5 studies), age (4 studies), and inhibin-B (3 studies). The results for high-response prediction for AFC were: cutoffs ≥14-16; Se=83.9%, Sp=8.9% (CI95% Se=75.2-91.0%, Sp=83.1-92.0%, 463 women), AUROC=0.84 (CI95%=0.77-0.92, 4,996 women). For AMH: cutoffs ≥3.0-5.0 ng/mL, Se= 78.4%, Sp=81.5% (CI95% Se=61.0-91.8, Sp=73.0-88.7%, 623 women), AUROC=0.83 (CI95%=0.79-0.88; 5,536 women). For basal FSH: cutoffs ≤2.0-7.0 UI/L, Se=63.6%, Sp=62.4% (CI95% Se=32.3-89.6%, Sp=55.4-69.0%, 253 women), AUROC=0.66 (CI95%=0.60-0.73; 4,786 women). For age: cutoffs <26.5years Se=42.2%, Sp=70.4% (CI95% Se=27.7-57.9%, Sp=60.3-79.2%, 143 women), AUROC=0.61 (CI95%=0.54-0.68; 4,786 women). For inhibin-B: AUROC=0.67 (CI95%=0.60-0.75, 389 women). Substantial heterogeneity (I^2^>50%) was observed in most of these analyses.

**CONCLUSIONS:** AMH and AFC show a good ability to predict a high-response to COS, whilst age, basal FSH and inhibin-B show poor performance. These estimates should be interpreted with caution due to the use of different cutoff points and the observed heterogeneity across studies.

## P-45. Impact of Assisted Hatching Laser in Human Assisted Reproduction in Different Age Groups

**K.H. Ribeiro^1^, V.N. Perez^1^, M.H. Ribeiro^1^, G.C. Silva^1^**

*^1^CLINIFERT - Centro de Reprodução Humana / Florianópolis - SC*

**OBJECTIVE:** To evaluate the effect of Laser Assisted Hatching (AH) in pregnancy and miscarriage rates of ICSI cycles in various age groups.

**MATERIAL AND METHODS:** A retrospective study of 765 ICSI cycles, conducted between 2008 and 2012, divided into two groups: 1. AH group with 435 cycles, 2. No Assisted Hatching (NAH) with 330 cycles. They were subdivided into four age groups: ≤ 30, 31-35, 36-39 and > 40 years. The criteria for inclusion in the AH group were presence of pellucid zone (ZP) thickness > 16 um or increased resistance, women aged > 37 years, elevated FSH and previous IVF failures. All other cases and embryos with normal ZP, independent of maternal age were included in the NAH group. Fresh embryos, Grade I and II, were transferred on day 3 in the AH group and on days 3 and 5 in the NAH group. AH was performed by the same operator using the OCTAX system, 2 to 4 pulses at a single point of ZP (about 10-12um). t Student and X2 with Yates correction tests were used for statistical analysis.

**RESULTS:** There was no difference between AH and NAH groups regarding the number of embryos transferred, fertilization, implantation and clinical pregnancy (*P* > 0.05). Only AH group with women < 30 years, the pregnancy rate was significantly higher compared to the NAH group (52.4% vs. 40.6% *P* < 0.05), with no differences in other age groups.

The miscarriage rate was significantly lower in the AH group compared to NAH group, at ages 36-39 years (25% vs. 41.8%) and > 40 years (33.3 vs. 53.9%), *P*<0.05.

**CONCLUSIONS:** Although many factors influence the success of a cycle of ICSI, the study suggests a beneficial effect of AH laser increasing pregnancy rates only in young women under 30 years.

However, the AH laser can be a good strategy in women over 35 years due to lower rates of miscarriages, with an favorable impact on clinical outcomes.

## P-46. Evaluation of the frequency of G-765C polymorphism in the promoter region of the COX2 gene and the correlation with the expression of this gene in the endometrium of women with endometriosis

**V. Cavalcanti^1^, T. Guida^1^, G. André^1^, D.M. Christofolini^1^, C.P. Barbosa^1^, B. Bianco^1^**

*^1^Center of Human Reproduction and Genetics - Department of Human Reproduction -Faculdade de Medicina do ABC, Santo André / SP, Brazil*

**OBJECTIVE:** To evaluate the frequency of the G-765C (rs20417) polymorphism of the COX2 gene and the expression of this gene in the endometrium of women with endometriosis.

**MATERIAL AND METHODS:** Case-control study with 375 women with endometriosis [251 infertile and 114 fertile] underwent laparoscopy/laparotomy with histological confirmation of the endometriosis. The control group consisted of 503 fertile women without endometriosis. Of these patients, 37 of the endometriosis group and 47 controls underwent endometrial biopsy for gene expression analysis. The genotypes were determined using a High Resolution Melt analysis. The gene expression was measured by qRT-PCR with TaqMan Gene Expression Assays and the GAPDH gene was used as a normalizing agent for the reactions.

**RESULTS:** The frequencies of genotypes and alleles between infertile and fertile endometriosis groups in relation to the control groups showed a trend towards significance in infertile endometriosis group, *P* = 0.074; OR=0.79; CI=0.61 to 1.02. The distribution of genotypes and alleles in relation to severity of disease found an association of the wild G allele as a protective factor for moderate/severe (*P*=0.028, OR=0.53, CI=0.32 to 0.90) fertile endometriosis. Considering the expression of COX2 in the group of women with endometriosis, the mean expression of COX2 was statistically higher when compared to the control, p= 0.028 and the minimal/slight endometriosis group when compared to the control group showed statistically higher expression of COX2, *P*=0.029.

**CONCLUSIONS:** This study found a positive association between the wild-765G allele and moderate/severe fertile endometriosis and an increased COX2 expression in eutopic endometrium of women with endometriosis when compared to the control group.

**Acknowledgment:** This study is supported by FAPESP (Fundação de Amparo à Pesquisa do Estado de São Paulo); #2012/03564-0 and CAPES (Coordenação de Aperfeiçoamento de Pessoal de Nível Superior).

## P-47. Prediction of Ovarian Hyperstimulation Syndrome: Systematic Review and Meta-Analysis

**C.O. Nastri^1^, R. Moroni^1^, V.M. Leitão^1^, D.M. Teixeira^1^,W.P. Martins^1^**

*^1^Setor de Reprodução Humana, Departamento de Ginecologia e Obstetrícia, Faculdade de Medicina de Ribeirão Preto, Universidade de São Paulo*

**OBJECTIVE:** Identify, appraise and summarize evidence regarding methods used for prediction of ovarian hyperstimulation syndrome (OHSS).

**MATERIALS AND METHODS:** Systematic review and meta-analysis of cohort studies evaluating methods to predict moderate/severe OHSS. The search was performed in the databases MEDLINE and Scopus. Sensitivity (Se), specificity (Sp), and area under the ROC curve (AUROC) were pooled and expressed with the 95% confidence interval (95%CI). Heterogeneity was evaluated by the I2.

**RESULTS:** Seven studies were included, comprising 261,879 women. The incidence of moderate severe OHSS in these studies was 3.8% (95%CI 2.3-5.6%). The methods assessed before controlled ovarian stimulation were: AFC, AMH, FSH, and age; each of them was examined by only one study. The results for AFC were: cutoff ≥ 23 follicles, Se=58.1%, Sp=74.7%, AUROC=0.74 (95%CI=0.670.82), 1,012 women. For AMH: cutoff > 3.36 ng/mL, Se=90.5%, Sp=81.3%, AUROC=0.90 (95%CI=0.86-0.93), 262 women. For age: cutoff < 33 years, Se=76.2, Sp=56.0%, AUROC=0.71 (95%CI=0.65-0.76), 262 women. The methods assessed on the day of hCG were: estradiol (3 studies), number of mid/large follicles (4 studies), and VEGF rise (1 study). For estradiol: cutoff > 1,430-2,560 pg/mL, Se=79.1%, Sp=65.2%, AUROC=0.75 (95%CI=0.66-0.84), 2,786 women. For mid/large follicles count: cutoff > 11-25 follicles, Se=87.9%, Sp=67.8%, AUROC=0.85 (95%CI=0.82-0.87), 3,410 women. For VEGF rise: cutoff ≥ 1 ng/mL, Se=100%, Sp=59.8%. Additionally, four studies assessed the number of retrieved oocytes to predict OHSS: cutoff ≥ 11-15, Se=73.1%, Sp=74.0%, AUROC=0.82 (95%CI=0.77-0.86) 256,643 women. Substantial heterogeneity (I^2^>50%) was observed in the analyses.

**CONCLUSIONS:** The most accurate and studied methods for predicting OHSS are the number of mid/large follicles on the day of hCG and the number of oocytes retrieved. Peak estradiol had only regular ability to predict SHO; while CFA, AMH, age and increased VEGF were evaluated by only one study each.

The estimates obtained should be interpreted with caution because studies used different cutoff points and also because of the observed heterogeneity across studies.

## P-48. Prediction of Metaphase II Oocytes According to Different Serum AntiMüllerian Hormone (AMH) Levels in Antagonist ICSI Cycles

**M.C.B. de Souza^1^, A.C.A. Mancebo^1^, J.B. da Silva^1^, R.A. Antunes^1^, M.M. de Souza^1^**

*^1^FERTIPRAXIS, Rio de Janeiro/RJ*

**OBJECTIVE:** AMH measurement has been proposed to predict quantitative and qualitative aspects in ART. Published studies seem to indicate it, prior to gonadotrophin secretion, as a better marker in predicting ovarian response to COS than age of the patient, Day 3 FSH, estradiol and inhibin B. Cut-off values aim to identify women at risk for poor-response or no response to gonadotrophins, thus contributing to reducing the cycle cancellation rate, the treatment costs and psychological stress for the couples.

Variable AMH predictive performance have been reported, partly atributed to different variants of assay. Now there are two types, produced by a single company (Beckman- Coulter), and cross-referencing has shown that their correlation is very high.

However, clinical application still depends on individual centers examining their own data, and so we aimed the correlation between AMH value and the ultimate ovarian response, that is, metaphase II oocytes.

**MATERIALS AND METHODS:** Observational study, 80 patients undergoing 82 antagonist ICSI cycles in a single center. Results were analyzed according to serum AMH: subgroup analyses were performed according to three AMH ranges: Group1- from 0,12 to 0,5 mcg/dL (44 cycles), Group 2-from 0,51 to 0.99 (25cycles) and Group 3-from 1 to 3(13 cycles).

Variables were checked as patient’s age, number of stimulation days, number follicles from 10-15mm and ≥16 on hCG day, n of aspirated oocytes and n of metaphase II oocytes. Statistical analysis was performed by ANOVA.

**RESULTS:** AMH seems to be a better marker in predicting ovarian response to controlled ovarian stimulation than age. There were significant difference between groups 1 and 3 regarding the ovarian response (n of follicles ≤15mm, ≥16 and MII oocytes recovered.

A high risk for no oocytes at pick-up was identified by serum AMH levels ≤0.5 mcg/dL (n =10.23%) versus 3(12%) in AMH from 0.51 to 9.99mcg/dL and none inAMH≥1.

**CONCLUSIONS:** From a practical standpoint, AMH is particularly useful to predict ovarian response to stimulation (MII oocytes), independently of the patient’s age, comparing the lowest value group to the normal one. We still lack confirmation on the 0.51-0.99 interval in order to the individualization of treatment strategies. Women with normal AMH levels are most probably normal responders and a good prognosis may be anticipated.

## P-49. Viability of Murine Blastocysts Vitrified in Macrovolume

**A.R. Batschauer^1^, V.L.L. Amaral^1^, M. Frajblat^2^, R.A. Salvador^1^, A. Senn^3^**

*^1^UNIVALI - Universidade do Vale do Itajaí - Itajaí, Santa Catarina*

*^2^UFRJ - Universidade Federal do Rio de Janeiro ^3^FABER - Fondation pour l’Andrologie, la Biologie et l’Endocrinologie de la Reproduction - Lausanne, Switzerland*

**OBJECTIVE:** Vitrification has great importance for the storage of surplus embryos and gametes, embryos and ovarian tissue banking, both in human and animal area. Usually, this technique relies on minimum volumes to achieve high cooling rates.

The aim of this study was to evaluate the efficiency of a protocol for vitrification of mouse blastocysts in macrovolume.

**MATERIALS AND METHODS:** The murine embryos were divided into three groups: control non vitrified (n=119), vitrified with Medium Kit for Vitrification of oocytes and embryos Ingámed^®^ into Vitri-Ingá strip (Ingámed^®^) (n=63) and vitrified in macrovolume (n=247), loaded into straws. In the macrovolume group, embryos were exposed for 3 minutes at VS1 (10% Propanediol 10% Ethyleneglycol, 0.25 M Sucrose, HTF modified (Irvine^®^) + 10% Fetal Bovine Serum - FBS), 30 seconds VS2 (20% Propanediol, 20% Ethyleneglycol, 0.5M Sucrose, HTF modified (Irvine^®^) + 10% FBS) loaded into 0.25 ml straws in a volume of 50µL, and immersed in liquid nitrogen.

On heating, the straws were kept in air at room temperature for 20 seconds, 40 seconds in water at 25°C, embryos were then transferred into solutions of 0.3M, 0.15M Sucrose, and HTF modified (Irvine^®^) + 10% FBS, for 5 minutes in each solution.

After warming, the embryos were kept in culture media Global (Lifeglobal^®^) + 10% FBS, at a temperature of 37°C and 6% CO2, for later verification of embryo survival and hatching. The results were analyzed by the χ2 (chi-square) test.

**RESULTS:** The survival and hatching rates were, respectively, 89.6% and 53% in macrovolume group, 93.1% and 84.1% in Ingaméd^®^ Kit group and 94.1% and 89.1 % in the control group.

**CONCLUSIONS:** The results of this study demonstrate that vitrification in macrovolume in straws maintains the integrity and viability of mouse blastocysts with good embryonic survival rates, however, with reduced hatching rate compared to the control group and Ingaméd^®^ Kit.

## P-50. Preimplantation Genetic Screening by Fluorescent Quantitative PCR (QF-PCR) in Blastocysts Embryos

**A.C.N. Martinhago^1^, N.C. Rugna^1^, K.R.N. Endo^1^, A.M.M. Dias^1^, G.P. Santos^1^, C.D. Martinhago^1^**

*^1^Chromosome Medicina Genômica - São Paulo/SP - Brasil*

**OBJECTIVES:** Select chromosomally normal embryos obtained by fertilization in vitro using the technique of QF-PCR - Quantitative fluorescent polymerase chain reaction (QF-PCR).

**MATERIALS AND METHODS:** All embryos were biopsied at the blastocyst stage and trofoectodermas cells (TE) were sent frozen to our center for further analysis. Cell lysis was performed in TE cells followed by the pre-amplification of the whole genome with a commercial kit. Polymorphic markers in the multiplex amplification reaction was performed in the polymerase chain reaction for 16 different chromosomes (X, Y, 3, 4, 5, 7, 11, 13, 14, 15, 16, 17, 18, 19, 21, 22). After amplification, fragments of different fluorescences were separated by capillary electrophoresis ABI3500 automatic sequencer and analyzed in GeneMarker and GeneMapper software.

**RESULTS:** During a year 638 embryos were evaluated. The main indications were exclusion of aneuploidy and advanced maternal age. The mean age of patients was 39 years. Of the 638 analyzed embryos 298 (47%) were normal and 317 (50%) had one or more chromosomal abnormalities. In only 3% occurred amplification failure due to artifacts of the technique itself. The most frequent alterations, independently of age of the patients were trisomy and monosomy 21, monosomy X and monosomy 18.

**CONCLUSIONS:** Addition to the use in preimplantation genetic diagnosis for detection of monogenic diseases, fluorescent-quantitative multiplex PCR (QF-PCR) is a promising strategy for preimplantation genetic screening (PGS) using the main chromosome analysis. The technique allows identifying the origin of the alleles and discarding the possibility of contamination and consequently the misdiagnosis.

QF-PCR seems not be a robust methodology as the NGS and Microarray, but it is an excellent option for the PGS to developing countries as low cost test.

## P-51. Analysis of the 24 Chromosomes In Human Blastocyst Using Next Generation Sequencing (NGS)

**A.C.N. Martinhago^1^, N.C. Rugna^1^, K.R.N. Endo^1^, A.M.M. Dias^1^, M.A. Oliveira^1^, C.D. Martinhago^1^**

*^1^Chromosome Medicina Genômica - São Paulo/SP - Brasil*

**OBJECTIVE:** Select chromosomally normal embryos obtained by fertilization in vitro using the technique of NGS - Next Generation Sequencing.

**MATERIALS AND METHODS:** During November and December, 2013, embryos that reached the blastocyst stage were biopsied and vitrified. The biopsy was performed on the fifth day of development in buffered medium with 10% Serum Protein Substitute. 5 to 10 trophoectoderm (TE) cells were removed. The collapsed embryos were vitrified. Biopsied TE cells were frozen in 3µL of PBS for analysis. After thawing at room temperature, the cells were subjected to cell lysis and whole genome amplification (WGA) with a commercial kit. The templates were prepared using barcodes for self-identification when simultaneous analysis of multiple samples were performed. Parallel sequencing of 32 embryos in a single chip occurred in Ion PGM™. After sequencing, analysis of the 24 chromosomes (22 pairs of autosomes and sex chromosomes X and Y) was taken in the Torrent Suite software ™ and Ion Reporter ™. The whole process was taken in no longer than 10 days.

**RESULTS:** 90 embryos were evaluated in total. The mean age of patients was 37 years and the main indications for PGS were exclusion of aneuploidy and advanced maternal age. It was possible to obtain results from all samples. 30 embryos analyzed (33%) were normal and 60 (67%) had one or more abnormalities. Despite the age of the patients, multiple changes and monosomy of chromosome 16 were the most frequent abnormalities observed, present in 8% of embryos. Monosomy of chromosome 1 was observed in 7% of embryos.

**CONCLUSIONS:** The technique of NGS for PGS combined with blastocysts biopsy and vitrification proved to be effective and robust. The main advantages of the technique are cost reduction and a high success rate analysis. Embryo screening by NGS technique proved to be a quick, accessible and promising methodology for clinical application in reproductive medicine for selecting embryos with the greatest potential for successful implementation.

## P-52. Peritoneal Fluid From Infertile Women with Minimal/Mild Endometriosis May Compromise the Spindle of Metaphase II Bovine Oocytes

**B.T.G.M. Jianini^1^, V.S.I. Giorgi^1^, H.Malvezzi^1^, M.G. Da Broi^1^, C.C.P. de Paz^2^, P.A.A.S. Navarro^1^**

*^1^Department of Obstetrics and Gynecology, Faculty of Medicine of Ribeirão Preto, University of São Paulo*

*^2^Department of Genetics, Faculty of Medicine of Ribeirão Preto, University of São Paulo*

**OBJECTIVE:** This study aimed to evaluate the impact of peritoneal fluid (PF) of infertile women with and without minimal/mild endometriosis (EI/EII) and fertile (fertile control; FC) on spindle integrity and chromosome alignment of bovine oocytes in vitro matured in the presence of PF.

**MATERIAL AND METHODS:** We performed an experimental study. PF samples were obtained from 12 women submitted to video laparoscopy (6 infertile women with EI/ II and 6 fertile controls - FC), which were utilized in 6 in vitro maturation (IVM) experiments of immature bovine oocytes (IBO). IBO were matured without PF (No-PF) and with three concentrations (1%, 5%, 10%) of two PF samples (EI/II and FC), totaling 10 groups. Each PF was used in one experiment. After 22-24 h of IVM, oocytes were denuded, fixed, stained for morphological visualization of both microtubules and chromatin, and analyzed by confocal microscopy. Data were analyzed by Poisson distribution.

**RESULTS:** The percentage of normal MII oocytes was significantly lower in the EI/II 1%, 5% and 10% concentrations (55.49 ± 0.04, 44.79 ± 0.04, 40.58 ± 0.05, respectively) compared to the same concentrations in the FC group (71.75 ± 0.04, 71.13 ± 0.03, 50.17 ± 0.04, respectively) and to the No-PF group (72.57 ± 0.03), *P*<0,01. There were no differences between FC and No-PF groups. In the EI/II group, we observed significantly lower percentage of normal MII oocytes in the concentrations 10% (40.58 ± 0.05) compared to 1% (55.49 ± 0.04) demonstrating a concentration-dependent effect. In the FC group, no differences regarding the concentrations were found.

**CONCLUSIONS:** FP of infertile women with EI/II promotes deleterious dose-dependent effect on spindle integrity and chromosome alignment of MII bovine oocytes in vitro matured. Thus this study opens a new perspective for understanding the pathogenesis of endometriosis-related infertility, suggesting that constituents of PF may be involved in the worsening of oocyte quality in women with EI/II. Financial funding: CNPq.

## P-53. The Odds Ratio for Tubal Occlusion, Ectopic Pregnancy, Hydrosalpinx and others Std in Infertile Chlamydia Trachomatis Seropositive Females

**F.C. Approbato^1^, M.S. Approbato^1^, M.C.S. Maia^1^, T.M. Silva^1^**

*^1^Human Reproductive Laboratory, Clinical Hospital, Federal University of Goias State, Brazil*

**OBJECTIVE:** The (+) C. trachomatis serology was associated to tubal obstruction (infertility), ectopic pregnancy (2nd cause of maternal death) and hydrosalpinx (infertility). It is a marker for others Sexually Transmitted Diseases (STD). Subject: Evaluate the Odds Ratio to tubal obstruction, ectopic pregnancy, hydrosalpinx and others STD at infertile seropositive patients for C. trachomatis.

**MATERIAL AND METHODS:** A Case-Control study.

Evaluation was done by IIF or IEE, C. trachomatis IgG, of 186 infertile patients, the Odds Ratio of 64 IgG (+), IIF or IEE patients, compared with 122 IgG (-). Variables: Odds Ratio for tubal obstruction, hydrosalpinx (HSG), ectopic pregnancy and others STD (Genital Herpes, HPV, Hepatitis B and Syphilis). We either did an evaluation of prevalence of C. trachomatis by PCR. The statistical test was the Chi-Square with Odds Ratio and 95 % C.I., *P*< 0.05.

**RESULTS:** It was not found statistical difference for: Tubal obstruction, hydrosalpinx and ectopic pregnancy.

It was found an increased risk for others STD in Chlamydia IgG (+) patients (Odds Ration 3.04; X2 = 4.94 *P*=0.026). It was found 2 PCR (+) patients, both at positive IIF / IEE group (Prevalence 1.04 %).

**CONCLUSIONS:** There was a 3.04 increase of prevalence for others DST when patients were sero (+) for C. trachomatis. It was not found prevalence increase for others variables. Chlamydia scarcely remains after teenager, when it is found only sequels.

## P-54. Pregnancy in Poor Responders after Oocytes Accumulation and Day Six Frozen-Thawed Embryo Transfer Cycle

**A.J.C. Meireles^1^, J.P. Bilibio^1^, F.C. Nascimento^1^, B.B. da Silva^1^**

*^1^Centro Especializado em Medicina de Reprodução Humana - Pronatus - Belém, PA*

**INTRODUCTION:** The oocytes accumulation by vitrification for a single cycle of IVF seems advantageous for poor responders. As well as blastocysts vitrification after prolonged culture can promote better selection and allows the embryo transfer at a more favorable moment.

**CASE REPORT:** Patient poor responder with 39 years old had three cycles of oocytes accumulation and vitrified 4MII and 1MI to be inseminated by ICSI with fresh oocytes from a next cycle. In cycle 4, frozen-thawed oocytes and a cool oocyte from the same cycle were injected. Semen concentration was 198x106/mL and 70% progressive motility. 18 hour after ICSI were found a frozen-thawed oocyte triploid and all the others with 2PN normal fertilization. On day 2, the fresh oocyte shown to be blocked and the remaining frozen-thawed oocytes continued with normal cleavages.

On day 5 just one compacted embryo showed only cavitation’s signals. For better definition of embryo quality and pregnancy outcomes, the group and patient decided to extend the culture until day 6. On day 6 there was a Blastocyst Hatching over 50% off the zona pellucida and how the patient didn’t have appropriate endometrium the group decided by freezing. Prior to aplicated the standard blastocysts vitrification protocol, it was completely removed from the zona pellucida by micromanipulation. After a new endometrium prepare, the blastocyst without zona pellucida was thawed and transfered after 3h of recovery. The patient became pregnant without intercourse during pregnancy that get to term with a healthy baby at home.

**COMMENTS:** It was observed that the oocytes accumulation by vitrification in poor responders can be quite effective since oocyte vitrification protocols are established with excellent survival rates. Similarly, blastocysts frozen at day 6 need to show morphology consistent with survival after frozen-thawed procedures, like good trophoectoderm cleavage and low fragmentation. Besides presenting visible inner cell mass and good prior cleavage rates as selection criterion.

## P-55. Fertility Preservation with Conservative Treatment Ectopic Pregnancy: A Case Report

**F.R.R. de Moraes^1^, E.L.V.T.R. de Moraes^2^, S.O. Pires^2^**

*^1^Supervisor of Medical Residency Program in Obstetrics and Gynecology, Federal University of Tocantins. ^2^Student of Medicine of Porto Nacional ITPAC*

**INTRODUCTION:** Ectopic pregnancy is when the embryo implants outside the uterine cavity, is a severe disease with high rates of maternal morbidity and mortality. The ectopic pregnancy has signs and physical findings like a intrauterine pregnancy. The association of the quantitative serum β-hCG with transvaginal ultrasonography optimizes the diagnosis of ectopic pregnancy. The treatment is divided into surgical, by salpingectomy and salpingostomy and clinician, who may be by medicines and expectant conservative. Drug treatment with methotrexate adopted in cases of ectopic pregnancy with integrity of the tube, minimizes the surgical and anesthetic risks, and provides a better reproductive prognosis.

**CASE REPORT:** 22/03/2005, the patient SNM, first pregnancy, 27 years old, with amenorrhea seven weeks and six days, sought medical attention complaining of abdominal pain associated with vaginal bleeding, the transvaginal ultrasound was identified a ruptured ectopic pregnancy, located to the right . She underwent laparotomy and right salpingectomy uneventful. 17/01/2006 the patient sought the same service, with amenorrhea seven weeks and two days, the ultrasound identified a not route ectopic pregnancy, with embryo no heartbeat, with CRL 12mm, quantitative β-hCG 82 mIU / ML. The patient underwent medical treatment with methotrexate in a single dose intramuscular dose of 50 mg / kg and showed very good therapeutic response with decrease in serum β-hCG. Due desire of a new pregnancy, was performed empirically, and with the intention of tubal patency, a HSG on 26/04/2006. This exam was evident an obstruction on the right fallopian tube, and the left was patent, but with tortuosity. In late 2006, the patient had an uneventful pregnancy topical, progressing to term, and in 2008 presented new topical pregnancy, also evolved to term.

**COMMENTS:** The clinical benefits of treatment with Methotrexate on Ectopic Pregnancy, established criteria for such, are widespread in the literature, this report highlights the use of hysterosalpingography after clinical treatment in order to preserve fertility by natural methods, favoring tubal patency, a fact noted in the case report.

## P-56. Comparison of Implantation Rates and Clinical Pregnancy Rates Between Biopsied Blastocists on Day 5 and 6 of Culture and Subjected at Cryopreservation and Subsequent Transfer

**K. Marques^1^, M.B. Bonavita^1^, J. Fioravanti^1^, B. Barros^1^, T. Criscuolo^1^, J.R. Alegretti^1,2^**

*^1^Center of Human Reproduction Hospital and Maternity Santa Joana - São Paulo*

*^2^Huntington Medicina Reprodutiva - São Paulo*

**OBJECTIVE:** Comparison of implantation and pregnancy rates of biopsied blastocists on Day 5 and 6 of culture, cryopreserved and, subsequent transferred.

**MATERIAL AND METHODS:** Between January/2013 and March/2014, were studied retrospectively a total of 191 biopsied blastocists (127 cycles) and were divided in 2 groups: A - Biopsied blastocists, cryopreserved, warmed and transferred on Day 5; B - Biopsied blastocists, cryopreserved, warmed and transferred on Day 6. Statistical analysis was performed based on X^2^ test and T test.

**RESULTS:** Joint analysis of the data observed that in the group A (embryos transferred on day 5) a clinical pregnancy rate of 62% and a implantation rate of 46.7%. In the Grup B (embryos transferred on day 6) was observed a clinical pregnancy rate of 45.7% and a implantation rate of 37.1%. Were not verified significant statistically difference between the two groups in the rate of clinical pregnancy (62.0% x 45.7%; *P*=0.09) and implantation rates (46.7% x 37.1%; *P*=0.31).

**CONCLUSIONS:** Ideal time to conducting the embryo biopsy at the blastocist stage is a point that must be respected, aiming to produce a smaller impact of the procedure. Thereby, the use of a blastocist biopsy on Day 6 of development showed similar rates of pregnancy and implantation in cycles wich used the vitrification as a cryopreservation method. The future transfer also in the same Day of biopsy is not statistically preliminary to the pregnancy and implantation rates. Thus, the present study demonstrated that late blastocists biopsies associated to the vitrification are options to be considerated routine in the laboratory.

## P-57. Distribution of Chromosomes Abnormalities Found in Blastocysts Submitted to Analysis by CGH- Array

**L. Luz^1^, A.L. Rossi^1^, R. Mazetto^2^, M. Tanada^2^, C. Brogliato^2^, J.R. Alegretti^1,2,3^**

*^2^Huntington Medicina Reprodutiva - Campinas*

*^2^Huntington Reproductive Medicine - São Paulo*

*^3^Department of Gynecology - Universidade Federal de São Paulo (UNIFESP) - São Paulo*

**OBJECTIVE:** Describe and discuss the incidence of chromosomal abnormality in blastocysts submitted to biopsy and analysis by CGH-array from In Vitro Fertilization cycles (IVF).

**MATERIAL AND METHODS:** From January to February 2014, 141 blastocysts obtained from IVF cycles were assessed and submitted to analysis by CGH-array. Patients with an advanced maternal age as well as the ones with previous implantation failure were included in IVF programme. After the result of aneuploidy, individual chromosomal errors such as monosomy and trisomy were evaluated individually for this study.

**RESULTS:** Embryo biopsy was performed on day 5 and 6 of the embryo development. Of the 141 blastocysts analyzed, 66% were assessed as aneuploid (93 embryos). Of the 93 aneuploid blastocysts, 66% were from patients aged 38 or more. Among the aneuploid embryos, 111 abnormalities were simple chromossomal loss (monosomy) and 99 simple gains (trisomy). Monossomies with high incidence were the ones of chromosomes 16 (12.61%), 21 (8.11%) and 22 (8.11%). Among trisomies the major incidence were the ones of chromosomes 16 (10.10%), 19 (8.08%) and 22 (10.10%). No trisomy of chromosome 5 was found.

**CONCLUSIONS:** The present study demonstrates that chromosomal errors occur almost equally in all chromosomes in both numerical loss and gains. However, less complexes technologies are not able to diagnose the embryo with the highest implantation potential. In addition, the idea of using only the cultivation to the blastocyst stage is not able to select chromosomally normal embryo or exclude the numerical errors in several chromosomes. These findings indicate that chromosome 16 is the responsible for major chromosomal abnormalities, similar to the existing published data. Indeed, preimplantacional diagnosis shows an important tool to identify the actual embryo viability in patients with advanced maternal age and previous implantation failures.

## P-58. Incidence of Monozygotic Twins in 1207 Cases after IVF Cycles

**M. Nicolielo^1^, A. Belo^1^, E. Semaco^1^, F. Mendez^1^, T. Criscuolo^1^, J.R. Alegretti^1,2^**

*^1^Huntington Reproductive Medicine - Sao Paulo*

*^2^Department of Gynecology Federal University of São Paulo (UNIFESP) - Sao Paulo*

**OBJECTIVE:** The aim of this study was to report the incidence of monozygotic twins in pregnancies coming from cycles of in vitro fertilization (IVF) from January 2011 to May 2013.

**MATERIAL AND METHODS:** Retrospective observational study conducted from January 2011 to May 2013. 1207 pregnancies from IVF cycles performed with the patient’s own eggs and transfers of fresh, thawed embryos cycles and performed with donor eggs were tabulated. All pregnancies were confirmed clinically by ultrassound and fetal heartbeat, 4-6 weeks after embryo transfer. The incidence of monozygotic was identified in cases where the number of gestational sacs visualized on ultrasound was greater than the number of embryos transferred.

**RESULTS:** Among the 1207 pregnancies evaluated in this study, 7 cases of monozigoze were observed and reported an incidence of 0.58%.

Among these, two were from cycles with fresh embryo transfer, two from frozen embryos and one from a cycle of egg donation. In all cases, the embryos were transferred on blastocyst stage.

**CONCLUSIONS:** The rate of monozigoze natural pregnancies is 1/90 (1.1%), and according to literature reviews, can vary from 0.7 to 13%, similarly found in our results, which was 0.58% in blastocyst transfers. Additional studies with large number of cycles can better identify and detail the bio-pharmacological events involved in order to minimize the multiplicity and its sequels.

## P-59. Use of Time-Lapse System in Euploid Blastocysts Identification (CGH-Array) on Fifth Day Embryo Development

**M. Bonavita^1^, J. Fioravanti^1^, K. Martins^1^, P. Serafini^1,2,3^, E.L.A. Motta^1,2,4^, J.R. Alegretti^1,2,4^**

*^1^Centro de Reprodução Humana Hospital e Maternidade Santa Joana - São Paulo*

*^2^Huntington Medicina Reprodutiva - São Paulo*

*^3^Centro de Reprodução Humana - Universidade de São Paulo (USP) - São Paulo*

*^4^Departamento de Ginecologia - Universidade Federal de São Paulo (UNIFESP) - São Paulo*

**OBJECTIVE:** To analyze the incidence of euploid blastocysts after trofectoderm biopsy, evaluated by comparative genomic hybridization on 5^o^ day embryo extended culture through morphokinetics blastocoele recovery, analysed by Time Lapse system.

**MATERIALS AND METHODS:** Twelve pre-blastocyst stage embryos were biopsied and subjected to analysis of trofectoderm cells by CGH-array on day 5 (D5) of embryonic development. They were then put back into cultivation triple gas and morphokinetics events evaluated in post-biopsy hours (hpb), as follows: a) early re-expansion of the blastocoel (TiR / hpb); b) full re-expansion of the blastocoel (TTR / hpb) and c) early hatching (TH / hpb) until day 6 (D6) using time-lapse (Primo Vision Embryo Monitoring System). The transfer of euploid embryos was performed on day 6 of cultivation.

Statistical analysis used was the t test and p values ≤ 0.05 were considered statistically significant.

**RESULTS:** Blastocysts were biopsied in an average of 128.7 ± 8.9 hours. The comparative analysis between the euploid and aneuploid blastocysts showed lower times in euploid embryos: early re-expansion of the blastocoel (9.2 ± 2.1 vs 17.0 ± 13.9 hpb, respectively, *P* = 0.436.) complete re-expansion (2.4 ± 1.4 x 4.5 ± 2.2 hpb, respectively, *P* = 0.278) and early hatching (3.8 ± 1.8 x 5.7 ± 2.1 hpb, respectively, *P* = 0.248) lower among euploid embryos. However, no statistically significant differences were found. From 12 pre-embryos analyzed, 9 were euploid and 3 were aneuploid.

**CONCLUSIONS:** These data suggest that pre-aneuploid embryos may have a slight delay in post-biopsy development comparing to pre-euploid embryos.

A better rate of post-biopsy development could also assist in choosing the most competent embryo, increasing process efficiency and enhancing a single embryo transfer. Due to the number os blastocysts analysed, a larger scale study is necessary.

## P-60. Excessive Ovarian Response to Stimulation with Pulmonary Thromboembolism

**L.M. Neves^1^, R.M.B. Scarabichi^1^, F.S. Tartari^1^, M. Cavagna^1^, A. Dzik^1^, N.F. Donadio^1^**

*^1^Hospital Pérola Byington - São Paulo - SP - Brazil*

**INTRODUCTION:** The Ovarian Hyperstimulation Sindome (OHSS) is an important complication of assisted reproduction, due to the morbidity and mortality. The release of vasoactive substances lead to loss of fluid to the third space and hemoconcentration. There is an increased incidence of thromboembolic events, and pulmonary embolism (PE) is the most severe manifestation.

**CASE REPORT:** Patient, 26 years old, white, healthy, with primary infertility for 5 years. Had irregular cycles without dysmenorrhea or dyspareunia. On examination: normotensive, weight 70 kg and BMI 24.2, discrete hirsutism, normal gynecological examination. Conducted three cycles of induction with clomiphene citrate unsuccessfully. The propaedeutics revealed normal hormonal level, AMH = 8.33ng/ml, basal ultrasonography with polycystic ovaries and hysterosalpingography with normal uterine cavity and patent tubes. Semen analysis was normal. It was set IVF protocol: FSHr 150 IU, cetrorelix 0.25 mg and 0.2 mg triptorelin to trigger. FSHr was suspended in E12 and introduced mini dose of hCG (200 IU). 11 MII oocytes were aspirated, and we chose to vitrify oocytes and embryos without transfer. The patient was readmitted on the same night with moderate OHSS. Laboratory tests revealed hemoconcentration (Hb = 14.7 / Ht = 47.8) and ultrasonography, moderate ascites. The patient was treated with hyperproteic diet, oral and IV fluids, albumin, symptomatic drugs and prophylactic enoxaparin. It was held strict control of diuresis and weight, ultrasonographic follow-up, blood count and electrolytes daily. The patient improved significantly and was discharged after three days. Returns after 9 days with sudden progressive dyspnea and chest pain. The D-dimer was 4556 and tomography of the chest showed a thrombus occluding branch of the right lower lobe artery, subsegmental atelectasis and bilateral pleural effusion, with no changes in Doppler of the lower limbs.

**COMMENTS:** Various strategies for preventing Ovarian Hyperstimulation Syndrome have been performed, however, there was progression to the severe form of the syndrome with pulmonary embolism. Much is said about the role of hCG as a mediator required for OHSS. This report and other relevant studies indicate the possible existence of other mediators in the pathophysiology of OHSS and point to the importance of strategies for prevention and treatment of the syndrome beyond the already established.

## P-61. Association Between Serum Anti-Müllerian Hormone Levels and IVF Outcomes

**M.F. Fortis^1,2^, M.A. Höher^1^, C.G. Basso^1,2^, N.P. Oliveira^1^, M.O. Ferreira^1^, N. Frantz^1^**

*^1^Center of Human Reproduction Nilo Frantz, Porto Alegre, RS, Brasil*

*^2^Federal University of Rio Grande do Sul, Porto Alegre, RS, Brasil*

**OBJECTIVE:** Verify the association between of anti-Müllerian hormone (AMH) levels and in vitro fertilization (IVF) outcomes.

**MATERIAL AND METHODS:** Data from 366 consecutive patients undergoing IVF treatment in 2013 was assessed. Patients’ ages, serum AMH, aspirated oocytes number, mature oocytes, embryos produced, embryo transference (ET) execution, and clinical pregnancy rates were compared. Percentiles were used for the comparison of serum AMH among patients. The continuous variables with normal distribution underwent analysis of variance (ANOVA) and Tukey post-hoc test. Discrete variables were evaluated by Chi-squared test and Fisher’s exact test. Statistical analysis was performed using SPSS version 20 software, differences were considered significant when *P*<0.05.

**RESULTS:** The patients’ average age was of 35.02 ± 4.42 years, the mean age of serum AMH was significantly different in p25-50 and percentiles higher than 90. Of the 3,145 oocytes retrieved, 2,690 (85.5%) were in MII stage. Oocytes were inseminated by ICSI, resulting in 1,942 embryos and a 76.03% fertilization rate. In 75.96% of the cycles the embryo transfer (ET) was performed, its occurrence was associated to p25-75 of AHM (*P*<0.01). Serum AHM above p75 was associated with ET cancelation (*P*<0.001) following total cryopreservation of the embryos (*P*<0.05) due to risk of ovarian hyper-stimulation syndrome (OHSS). The clinical pregnancy rate per ET was of 42.4%, with no significant difference between different serum AMH percentiles.

**CONCLUSIONS:** AMH is an excellent tool for the evaluation of ovarian reserve and a good predictor of ovarian response. This work suggests that AMH is capable of predicting the cancelation of embryo transfers. Patients whose serum AMH is above the 75th percentile presented high risk of OHSS, therefore the safest strategy for their treatment consists in embryo cryopreservation and shortterm delayed ET.

## P-62. Genetic Study of First Trimester Pregnancy Loss

**M.F. Fortis^1,2^, C.G. Basso^1,2^, N.P. Oliveira^1^, F.K. Robin^1^, S. Mattiello^1^, N. Frantz^1^**

*^1^Center of Human Reproduction Nilo Frantz, Porto Alegre, RS, Brasil*

*^2^Federal University of Rio Grande do Sul, Porto Alegre, RS, Brasil*

**OBJECTIVE:** Investigate the karyotypes of miscarriage specimens.

**MATERIAL AND METHODS:** In the time between June 2012 and May 2014, 29 miscarriage specimens from 24 couples were acquired through manual intra-uterine aspiration (MIUA) in our human reproduction clinic. The villi samples were carefully identified and placed in saline and were shipped for the karyotype exam to be performed. RESULTS: The patients average age was 35,58 years (varying between 30 and 41 years of age). Seven patients have had at least one previous pregnancy loss, seventeen had none miscarriages. Five out of twenty-four pregnancies (20.8%) were multiple, adding up to 29 karyotypes performed. Four couples had exclusive male factor infertility, nine were infertile due to exclusive female factor and four presented male and female causes for infertility. Seven couples were fertile and got pregnant spontaneously. All abortions occurred between six and twelve weeks of pregnancy, the most common interruption age was 8 weeks of pregnancy. The karyotype analysis revealed that 44.8% (13/29) of the specimens had abnormalities, 34.5% (10/29) were normal female, 17.2% (5/29) normal male, and in 3.5% (1/29) there was no cellular growth and, therefore, no result was obtained. Of the normal karyotypes, 66.6% were female and 33.3% were male.

**CONCLUSIONS:** Cytogenetic abnormalities represent an important cause of pregnancy loss and its detection helps in couples’ genetic counseling. The main causes of miscarriage found were chromosomic anomalies, represented mainly by trisomy, polyploidy and monosomy of the X chromosome. Evidence of chromosomic abnormalities was found in 44.8% of the miscarriage specimens examined. Prior studies suggest that 45 to 70% of first trimester miscarriages are caused by chromosomic anomalies. Even though this study shows that we have a low rate of cellular growth failure in karyotype of miscarriage specimens, due to the overrepresentation of the 46,XX karyotype, these results should be interpreted carefully. Lathi *et al*. have shown over half of the 46,XX results in miscarriage are due to maternal cell contamination. Therefore, molecular karyotyping techniques, such as SNP array, must be adopted in order to improve the accuracy of products of conception testing.

## P-63. Shared Pregnancy in Homoaffective Couple: A Case Report

**M.C.S. Maia^1^, M.S. Approbato^1^, T.M. Silva^1^, C.R. Giviziez^1^, E.A.B. Fleury^1^, M.S. Ramos^1^**

*^1^Laboratório de Reprodução Humana, Hospital das Clínicas, Universidade Federal de Goiás*

**INTRODUCTION:** Every homoaffective couple seeking assisted reproduction clinics to have their children. The Federal Council of Medicine published a new resolution in 2013 which ensure couples including same-sex the right to appeal to the Human Reproduction to have children.

**CASE REPORT:** During the second half of 2013, MAGP, 35 years and TCAP, 29 years, homoafetivo couple sought our service with the objective of achieving pregnancy using donor semen. After conducting routine examinations the following induction was performed for TCAP, recipient of the embryo: Primogyna 1 mg 12/12 hours from 1st to 4th day, primogyna 2 mg 12/12 hours from the 5th to the 9th day and 8/8 hours 10th to 14th day and 3 mg primogyna 8/8 hours on day 15. The induction MAGP, donor oocytes: Clomiphene citrate 100 mg the 2nd to 6th day, rFSH (150 IU / day) from the 3rd to 15th day, rLH 75 IU of the 10th to 15th day, Cetrotide 12th to 16 th and r-hCG (250 ug) on the 16th day. Evolved with three follicles 17 mm showed that on the 15th day of the cycle. Follicle puncture was performed and captured an oocyte in metaphase II, and then performed ICSI. On the 3rd day of culture was transferred to receiving an embryo with 8 cells. After 14 days, the dosage of β-HCG was 197.4 mIU /ml. The patient returned to performing USG, with gestational age of 7 weeks, one gestational sac and BCF gifts. The pregnancy was full term with the birth of a female child in April 2014 at 39 weeks and weight 3,300 Kg.

**COMMENTS:** This case report illustrates that the right to procreation belongs to everyone and is recognized in the Universal Declaration of Human Rights. At present, the techniques of assisted reproduction represent a real alternative to homosexual couples achieve maternity, bringing our services to new demand and new perspectives.

## P-64. Effects of Recombinant Luteinizing Hormone Supplementation During Controlled Ovarian Stimulation in IVF/ICSI Cycles

**M.C.S. Maia^1^, M.S. Approbato^1^, T.M. Silva^1^, E.A.B. Fleury^1^, M.Z.P. Brito^1^, C.C. Aguiar^1^**

*^1^Laboratório de Reprodução Humana, Hospital das Clínicas, Universidade Federal de Goiás*

**OBJECTIVE:** To compare the effects of recombinant LH (r-LH) supplementation for controlled ovarian stimulation with recombinant FSH (r-FSH) in the GnRH antagonist protocol in IVF/ICSI cycles.

**MATERIAL AND METHODS:** A case-control study was performed of 88 patients, aged between 34-42 years, classified into two groups according to an ovarian stimulation scheme: Group I (n= 38): r-FSH + r-LH and Group II (n= 50): r-FSH (control group). The following variables were paired: age, duration of infertility, serum FSH, LH and estradiol. Number of oocytes retrieved, number of oocytes in metaphase II, fertilization rate, implantation rate and rates of chemical and clinical pregnancy were analyzed. Differences in proportions were evaluated by chi-square test and medians by Wilcoxon Mann-Whitney test, a significance level of *P*<0.05. Data analysis was performed using Bioestat 5.3^®^ software.

**RESULTS:** The median age of patients in Group I was 37.8±2.7 and Group II 36.6±2.6 (*P*=0.08). An equal distribution (*P*>0.05) of the other variables was also observed. There were no significant difference between the two groups regarding: number of oocytes retrieved (3.7±2.2 x 3.9±1.4, *P*=0.07), number of oocytes in metaphase II (2.5±1.7 x 2.7±1.5, *P*=0.20), fertilization rate (78.9% x 70.0 %, OR 1.60, 95% CI 0.59-4.31, *P*=0.48), implantation rate (19.6% x 14.8%, OR 1.36, 95% CI 0.54-3.44, *P*=0.66), rate of chemical pregnancy (23.7% x 20.0%; OR 1.24, 95% CI 0.44-3.44, *P*=0.87) and clinical pregnancy rate (21.0% x 16.0%, OR 1.40, 95% CI 0.47-4.14, *P*=0.74).

**CONCLUSIONS:** In this study it was concluded that supplementation with r-LH showed no benefit with regard to variables during controlled ovarian stimulation with GnRH antagonists.

## P-65. Hyaluronan-Binding System For Sperm Selection Enhances Pregnancy Rates in ICSI Cycles Associated with Severe Male Factor

**R.F. Erberelli^1^, S.S. Silva^1^, R.M. Salgado^1,2^, A.C. Mathias^1^, J.G. Aguiar^1^, P. Wolff^1,2,3^**

*^1^Clinica Genics - Medicina Reprodutiva e Genômica*

*^2^ Reprolab - Manaus*

*^3^Invitrogenese - Biologia do Desenvolvimento e Rep. Assistida*

**INTRODUCTION:** The aim of the present study was to compare two procedures for sperm selection in IVF cycles - conventional ICSI and Hyaluronan-treated petri dishes (PICSI), when severe male factor was associated. The evaluated parameters were: fertilization and cleavage rates, chemical and clinical gestation rates, as well as abortion rate.

**CASES REPORT:** Fifty-six ICSI cycles were included in this report, 19 cycles using PCSI dishes and 37 using conventional ICSI. Respectively, fertilization rates were 71,42% (123/171) and 64,14% (127/198); cleavage rates were 95,98% (117/123) and 95,27% (121/127); chemical pregnancy rates were 63,15% (12/19) and 27,06% (10/37); clinical pregnancy rates were 42,10% (8/19) and 16,21% (6/37); and abortion rates were 33% (4/12) and 40% (4/10). *P* values ≤ 0.05 were considered statistically significant. According to the Fisher’s Exact Test, only chemical pregnancy rates were significantly different (*P*<0.01). Clinical pregnancy rates show strong tendency to statistical significance favoring PICSI (*P*=0.051).

**COMMENTS:** Our preliminary results indicate that there are differences in pregnancy rates. The Hyaluronan-binding system mimics the cumulus oophorus extracellular matrix to induce binding of sperm head to the petri dish. Sperm cells presenting CD44 (Hyaluronan receptor) on the head membrane are considered mature, with high acrossomal and mitochondrial integrity. On the other hand, immature sperm are considered unable to bind to Hyaluronan. They retain cytoplasmic enzymes that cause peroxidation, inducing DNA fragmentation and sperm morphology alterations.

## P-66. Comparative Analysis of the Results of in Vitro Fertilization Between two Protocols Involving Culture Media and the Day of the Embryo Transfer

**R.R. Costa^1^, C.G. Almodin^1^, B.M. Silva^1^, N.I.Z.T. Cabral^1^, V.E.T. Sousa^1^, Y.A. de Macedo^1^**

*^1^Hospital Materno Infantil de Brasília - HMIB*

**OBJECTIVE:** compare success rates in IVF treatment of patients attended in public hospital of the DF after changes in treatment protocol involving the day of embryo transfer and embryo culture media used.

**MATERIAL AND METHODS:** retrospective study to evaluate comparatively positive biochemical pregnancy rates in patients with infertility in IVF treatment analyzing two protocols involving the embryonic transfer day and the culture medium used. Of the 128 IVF cycles analyzed, 70 were guided using the Protocol A (transfer in D1-D3 of embryos grown in single-step media culture produced by Irvine Scientific). The other 58 cycles have been guided by the Protocol B (transfer in D4-D6 of embryos grown in single-step culture media produced by Ingamed). RESULTS: the Protocol A resulted in 17 positives beta-HCG’s (24.3%), against 26 of Protocol B (44.8%).

**CONCLUSIONS:** based on the data presented, the realization of embryonic transfer in D4-D6 using culture media of Ingamed showed better results in IVF treatments compared with embryos grown up to D1-D3 using culture media of Irvine Scientific.

## P-67. Sperm Selection by Different Techniques for Processing Seminal

**D.C. Cipriani^1^, V.B. Ramos^1^, A. Senn^2^, V.L.L. Amaral^1^**

*^1^UNIVALI - Universidade do Vale do Itajaí - Itajaí, Santa Catarina*

*^2^FABER- Fondation pour l’Andrologie, la Biologie et l’Endocrinologie de la Reproduction - Lausanne, Switzerland*

**OBJECTIVE:** The aim of this study was to evaluate the effectiveness of three methods for semen processing, Swim-up and Sedimentation (SS) using a Jundet chamber, Swim-up from fresh sample (SF) and Swim-up from washed sample (SL), for the selection of a larger number of motile sperm.

**MATERIALS AND METHODS:** Samples of 12 normozoospermic men were selected, divided into three equal aliquots and processed by the three techniques simultaneously. For washing in SL, the modified HTF + 10% SSS (Irvine^®^) was used, the same as the one used in SF and SS. The proportions used for SF and SL was 1 ml of medium for 1 ml fresh or resuspended sperm. SS test was performed using 1 ml of semen and 2.5 ml of HTF medium. The migration time at 37°C was1h for SF and SL and 1:30 for SS. At the end of migration, 200 µl were removed from the top of the supernatant (SF, SL) and the bottom of the central cone (SS) and analyzed for concentrations (Neubauer) and motility (slide / cover slip) of progressive, non- progressive and immotiles.

**RESULTS:** There were differences in mean recovery rates of the number of spermatozoa (*P*=0.01), the SS technique was higher (19.3%) compared to SF (6%) and SL (4.4%), but these last two were not different. Progressive motility was significantly different between SF and SL (*P*<0.001) and SL and SS (*P*<0.001), equally so for the non-progressive motility of SF and SL (*P*<0.05) and SL and SS (*P*<0.001). When comparing total motility there were also differences between the same techniques, SF and SL (*P*<0.01) and SL and SS (*P*<0.001). Finally there was no difference between the total motility using SF and SS techniques.

**CONCLUSIONS:** The SS technique in the Jundet chamber allows a recovery of a higher number of sperm compared to the techniques SF and SL. For the recovery of spermatozoa with progressive motility, SS and SF were the most effective, suggesting that the washing process in SL can damage the recovery of spermatozoa with progressive motility.

## P-68. Vitrification of Zygotes at the Pronuclear Stage: An Alternative for Cryopreservation?

**R.A. Salvador^1^, D. Til^1^, A. Senn^2^, V.L.L. Amaral^1^**

*^1^UNIVALI - Universidade do Vale do Itajaí - Itajaí, Santa Catarina*

*^2^FABER- Fondation pour l’Andrologie, la Biologie et l’Endocrinologie de la Reproduction - Lausanne, Switzerland*

**OBJECTIVE:** Vitrification is possible in different embryonic stages, but is differing in relation to protocols and survival rates. The aim of this study was to evaluate the efficiency of cryopreservation of murine pronuclear stage zygotes through vitrification.

**MATERIALS AND METHODS:** Zygotes were obtained from F1 (BALB / c X C57BL / 6) mice. Females were hyperstimulated with 10 IU of equine chorionic gonadotropin (Novormon^®^ - Syntex) and after 48 hours were administered 10 IU of human chorionic gonadotropin (Vetecor^®^ - Calier). After 16 hours the zygotes (n = 32) were collected and cryopreserved according to Ingámed^®^ protocol, by exposing them to the equilibrium VI-I solution for 18 minutes, transferring them to vitrification solution II-VI and putting the mon vitrification strips (n = 8/strip). The time between exposure to VI-II solution and immersion in liquid nitrogen did not exceed 60 seconds. Warming of the zygotes was performed by dipping the strips directly into DV-I solution preheated to 37ºC, where they remained for 1 minute before being transferred to the DV-II solution. After 3 minutes they were washed 2 times in DV-III solution for 5 minutes each. The zygotes were cultured in 30µL SSM droplets (Single Step Medium - Irvine^TM^) supplemented with 10% serum (SSS Serum Substitute Supplement - Irvine Scientific™) at a controlled temperature of 37° C and 5% CO_2_. Embryonic development was evaluated until the expanded blastocyst stage. A control group (n = 10, not cryopreserved zygotes) was maintained in culture conditions identical to the vitrified group.

**RESULTS:** In the vitrified group, the survival and blastocyst rates were respectively 91% (30/33) and 100% (30/30), for the control group the blastocyst rate was100% (10/10).

**CONCLUSIONS:** Our results demonstrate that vitrification allows the survival zygotes and development up to the blastocyst stage, allowing thus successful cryopreservation of a stage prior to syngamy or cleavage.

## P-69. The Evaluation of Devitrified Blastocysts Survival Rate and Clinical Outcomes According to their Morphology on Day 3

**I.H. Yoshida^1^, P.C. Rodrigues^1^, F.E. Mizrahi^1^, S. Glina^1^, L.O. Tso^1^, N.E. Busso^1^**

*^1^Projeto ALFA - Aliança de Laboratórios de Fertilização Assistida*

**OBJECTIVE:** The aim of the study was to evaluate the devitrified blastocysts survival rate and clinical outcomes (implantation and clinical pregnancy rates) according to their different embryonic morphologies on D3.

**MATERIALS AND METHODS**: Retrospective cohort study. The study has added exceeded embryos that has reached blastocyst stage and were vitrified from couples who underwent IVF/ICSI cycles from January 2010 to April 2014. Those were thawed in order to perform a new treatment and so they were divided into two homogeneous groups: G1: blastocysts that were classified as good quality on cleavage stage (n = 71) and G2: blastocysts that were classified as poor quality (n = 86). A total of 157 frozenthawed blastocysts were included in this research. The freeze-thaw protocol was the same for both groups using Cryotop^®^ open system and Irvine Scientific^®^ vitrification and devitrification medium. Some embryos transfers were made with more than an blastocyst, however we have only included in the study the transfers that showed all the blastocysts derived of the same morphological classification on D3. The implantation rate was considered as the number of gestational sacs divided by the number of embryos transferred. The clinical pregnancy rate was defined by the presence of the heartbeat on the eighth week of gestation. These data were correlated among the groups through Chi-Square test.

**RESULTS:** There was no significant difference between G1 and G2 when compared to the survival rate (85.92% vs. 82.56%) (*P*=0.564), implantation rate (38.02% vs. 30.23%) *(P*=0.303) and clinical pregnancy (40.84% vs. 32.55%) (*P*=0.281).

**CONCLUSIONS:** It was evaluated that the embryo quality on D3 does not affect survival rate and clinical outcomes when it reaches the blastocyst stage and so it is vitrified.

## P-70. Using Stretched Microcapillary to Reduce the Volume of Cryoprotectant During Vitrification of Mouse Embryos

**E. Pessiquelli^1^, R.A. Salvador^1^, V.L.L. Amaral^1^, C.M. Mendes^2^**

*^1^UNIVALI - Universidade do Vale do Itajaí/SC - Laboratório de Biotecnologia da Reprodução*

*^2^USP - Universidade de São Paulo/SP - Departamento de Reprodução Animal*

**OBJECTIVE:** The vitrification of embryos has become essential in routine clinical practice of assisted human reproduction. For the success of this technique it is critical to remove as much of the vitrification solution volume as possible, to prevent its toxic effect and speed up the cooling system. This study aims to use a glass stretched microcapillary to reduce the volume of vitrification solution and evaluate the effect on survival rates and embryo development after vitrification.

**MATERIALS AND METHODS:** 30 female mice F1 (C57Black6xBalb / c) aged 8 weeks were used. They were stimulated with 10 IU eCG and after 48 hours were administered 10 IU of hCG. Embryos were collected at 2 cells stage, and divided into four experimental groups. Control group - CTR, with fresh non vitrified embryos, G1, G2 and G3 with embryos vitrified using the vitrification kit and the strips Vitri-ingá® (Ingámed), while in G2 embryos were vitrified with an excess volume of vitrification solution and in G3 with a reduction of volume of the vitrification solution using a stretched glass microcapillary. Statistical analyzes were performed using the chi-square test, considering statistical significance with P<0.05.

**RESULTS:** Embryo survival rates (G1 83.3%, G2 77.7%, G3 71%), cleavage rates (100% G1, 95.2% G2, G3 90.9%) blastocyst formation (G1 68%, 65% G2, G3 80%) and hatching rates (100% G1, G2 92.3%, 93.7% G3) were not different between groups.

**CONCLUSIONS:** Although results were not different between groups, the greater ease of handling for removal of excess medium when using the stretched microcapillary indicates that this procedure may be another option for the vitrification technique.

